# VP3 protein of Senecavirus A promotes viral IRES-driven translation and attenuates innate immunity by specifically relocalizing hnRNPA2B1

**DOI:** 10.1128/jvi.01227-24

**Published:** 2024-08-29

**Authors:** Lu Li, Xinwei Li, Han Zhong, Mingyang Li, Bo Wan, Wenrui He, Yuhang Zhang, Yongkun Du, Dongjie Chen, Wei Zhang, Pengchao Ji, Dawei Jiang, Shichong Han

**Affiliations:** 1 International Joint Research Center of National Animal Immunology, College of Veterinary Medicine, Henan Agricultural University, Zhengzhou, China; 2 Longhu Laboratory, Henan Agricultural University, Zhengzhou University, Zhengzhou, China; 3 Ministry of Education Key Laboratory for Animal Pathogens and Biosafety, Henan Agricultural University, Zhengzhou, China; 4 Institute of Animal Inspection and Quarantine, Chinese Academy of Inspection and Quarantine, Beijing, China; 5 State Key Laboratory for Animal Disease Control and Prevention, Lanzhou Veterinary Research Institute, Chinese Academy of Agricultural Sciences, Lanzhou, China; St. Jude Children's Research Hospital, Memphis, Tennessee, USA

**Keywords:** Senecavirus A, hnRNPA2B1, translational control, IRES-driven translation, host shutoff, immune evasion

## Abstract

Viruses deploy sophisticated strategies to hijack the host’s translation machinery to favor viral protein synthesis and counteract innate cellular defenses. However, little is known about the mechanisms by which Senecavirus A (SVA) controls the host’s translation. Using a series of sophisticated molecular cell manipulation techniques, heterogeneous nuclear ribonucleoprotein A2B1 (hnRNPA2B1) was identified as an essential host factor involved in translation control in SVA-infected cells. It was also determined that the SVA structural protein, VP3, binds to and relocalizes hnRNPA2B1, which interferes with the host’s protein synthesis machinery to establish a cellular environment that facilitates viral propagation via a two-pronged strategy: first, hnRNPA2B1 serves as a potent internal ribosome entry site (IRES) *trans*-acting factor, which is selectively co-opted to promote viral IRES-driven translation by supporting the assembly of translation initiation complexes. Second, a strong repression of host cell translation occurs in the context of the VP3-hnRNPA2B1 interaction, resulting in attenuation of the interferons response. This is the first study to demonstrate the interaction between SVA VP3 and hnRNPA2B1, and to characterize their key roles in manipulating translation. This novel dual mechanism, which regulates selective mRNA translation and immune evasion of virus-infected cells, highlights the VP3-hnRNPA2B1 complex as a potential target for the development of modified antiviral or oncolytic reagents.

**IMPORTANCE:**

Viral reproduction is contingent on viral protein synthesis, which relies entirely on the host's translation machinery. As such, viruses often need to control the cellular translational apparatus to favor viral protein production and avoid host innate defenses. Senecavirus A (SVA) is an important virus, both as an emerging pathogen in the pork industry and as a potential oncolytic virus for neuroendocrine cancers. Here, heterogeneous nuclear ribonucleoprotein A2B1 (hnRNPA2B1) was identified as a critical regulator of the translational landscape during SVA infection. This study supports a model whereby the VP3 protein of SVA efficiently subverts the host’s protein synthesis machinery through its ability to bind to and relocalize hnRNPA2B1, not only selectively promoting viral internal ribosome entry site-driven translation but also resulting in global translation shutdown and immune evasion. Together, these data provide new insights into how the complex interactions between translation machinery, SVA, and innate immunity contribute to the pathogenicity of the SVA.

## INTRODUCTION

Senecavirus A (SVA), previously designated as the Seneca Valley virus, is a prototypic member of the genus *Senecavirus* within the family *Picornaviridae*. As a cytoplasmic RNA virus, SVA contains a positive-sense single-stranded genome of approximately 7.3 kb in length, which is composed of the 5′ untranslated region (5′-UTR), an open reading frame (ORF) encoding a single polyprotein, and the 3′-UTR. During SVA replication, the polyprotein is subsequently processed into VP4 structural proteins VP4, VP2, VP3, and VP1 and non-structural proteins L, 2A, 2B, 2C, 3A, 3B, 3C, and 3D by virus-encoded proteases (1). SVA is an emerging pathogen in swine that has been reported in numerous pork-producing countries, causing vesicular disease and neonatal mortality (2–4). SVA is also an oncolytic virus with selective tropism for neuroendocrine cancers (5, 6), and is under development in clinical trials as a therapeutic agent against these tumors (7, 8).

Viral reproduction is contingent on viral protein synthesis, which fully depends on the host’s translation apparatus. Consequently, viruses have evolved remarkable strategies to commandeer cellular protein synthesis machinery of the host, which not only preferentially favors viral protein production but also suppresses host gene expression to stifle innate host defenses. Usually, these processes involve the action of multi-functional virulence factors that secure the recruitment of ribosomes toward viral mRNAs, but not the host’s antiviral genes (9–11). A well-known example is the Nsp1 protein, a potent virulence factor of severe acute respiratory syndrome coronavirus (SARS-CoV) and SARS-CoV-2 that induces profound translational shutdown and immune evasion to facilitate viral replication (12–15). Furthermore, translational control in virus-infected cells has been proposed as a critical step in viral propagation with important effects on virulence, tissue tropism, and pathogenicity (9–11). Therefore, a better understanding of the complex virus-host translational landscape could have important implications for the development of effective antiviral strategies or oncolytic reagents.

It is common for RNA viruses to target the initiation step of the host protein translation system to preferentially or exclusively synthesize viral proteins (16). Unlike the canonical cap-dependent translation of most eukaryotic mRNAs, translation initiation in picornavirus RNAs occurs via a different mechanism. The 5′-UTR of all picornavirus genomes contains an internal ribosome entry site (IRES), which is characterized by numerous stem loops and pseudoknots, allowing internal binding of the ribosomes and subsequent translation initiation through multiple RNA-RNA and RNA-protein interactions (17–20). Notably, IRES-driven translation can proceed when the dominant cap-dependent translation is shut down (19); therefore, it is speculated that picornaviruses can take advantage of this to subvert the host translation machinery to favor viral protein synthesis and suppress the host’s defenses. However, the complex interactions between translation machinery, picornaviruses, and innate immunity remain poorly understood.

Notably, many proposed that the IRES *trans*-acting factors (ITAFs), belonging to RNA-binding protein (RBP) families, have been reported to play critical roles in determining the efficiency of viral IRES-driven translation initiation. ITAFs may function as RNA chaperones and cooperate with other initiation factors to maintain the proper IRES conformation for ribosome assembly (17, 19, 21, 22). Thus, the ITAF modulation of IRES-driven translation represents an alternative strategy for viruses to commandeer cellular translation resources during infection. For example, during infection with foot-and-mouth disease virus (FMDV), the ITAF nucleolin was found to relocalize from the nucleus to the cytoplasm where it promoted viral IRES-driven translation by supporting the assembly of translation initiation complexes (23). In addition, several novel ITAFs, including Sam68, FBP1, and SRp20, have been utilized by picornaviruses to enhance viral IRES activity (24–27). However, the molecular mechanisms by which ITAFs regulate SVA IRES-driven initiation of translation remain largely unknown.

Heterogeneous nuclear ribonucleoprotein A2B1 (hnRNPA2B1) is a multi-functional host RBP that is implicated in multiple stages of RNA processing, including transcription, alternative splicing, mRNA stability, decay, trafficking, translation, and even innate immunity (28). Owing to its crucial role in viral RNA processing and innate immunity, hnRNPA2B1 has also been shown to promote the replication of several RNA viruses (29–34). The current study aimed to elucidate the mechanisms by which ITAFs regulate SVA IRES-driven translation. Cellular RBPs that are associated with SVA 5′-UTR were systematically examined, as well as those translocated to the cytoplasm after SVA infection, resulting in the identification of hnRNPA2B1 as a novel ITAF for SVA IRES-driven translation. Functional analysis further revealed that hnRNPA2B1 was redistributed from the nucleus to the cytoplasm by binding with the SVA VP3 protein, and that cytoplasmic hnRNPA2B1 specifically supports viral IRES-driven translation initiation. Moreover, SVA VP3 also targeted hnRNPA2B1 to function in the host translational shutdown and immune evasion. Hence, this study uncovers a previously unknown dual role of hnRNPA2B1 in translational control during virus-host interactions.

## RESULTS

### Identification of hnRNPA2B1 as a positive regulator of SVA infection

To identify potential RBPs involved in SVA replication, a biotinylated RNA pull-down assay, followed by mass spectrometry (MS) was performed to examine the cellular proteins associated with the SVA 5′-UTR of SVA (Fig. S1A). In addition, to systematically identify cellular proteins that relocalize from the nucleus to the cytoplasm during SVA infection, mock- and SVA-infected cells were fractionized into their nucleic and cytoplasmic components which were subjected to MS to quantify the proteins (Fig. S1B). By integrating these two approaches, a highly selective re-localization of nuclear proteins with known viral RNA processing functions, including hnRNP K, nucleolin, and hnRNPL, were discovered (23, 35, 36). The presence of these RBPs supported the validity of our experimental approach. Notably, hnRNPA2B1 had 47 peptide matches, 62% coverage, and an Unused ProtScore of 238.75 in the MS data of the SVA 5′-UTR interaction proteomics (Table S1). According to the relocalized MS data, the abundance of hnRNPA2B1 was consistently increased in the cytoplasm (abundance ratio of SVA:mock, 4) and decreased in the nucleus (abundance ratio of SVA:mock, 0.82) at 8 h post-infection (hpi) (Table S1).

**Fig 1:**
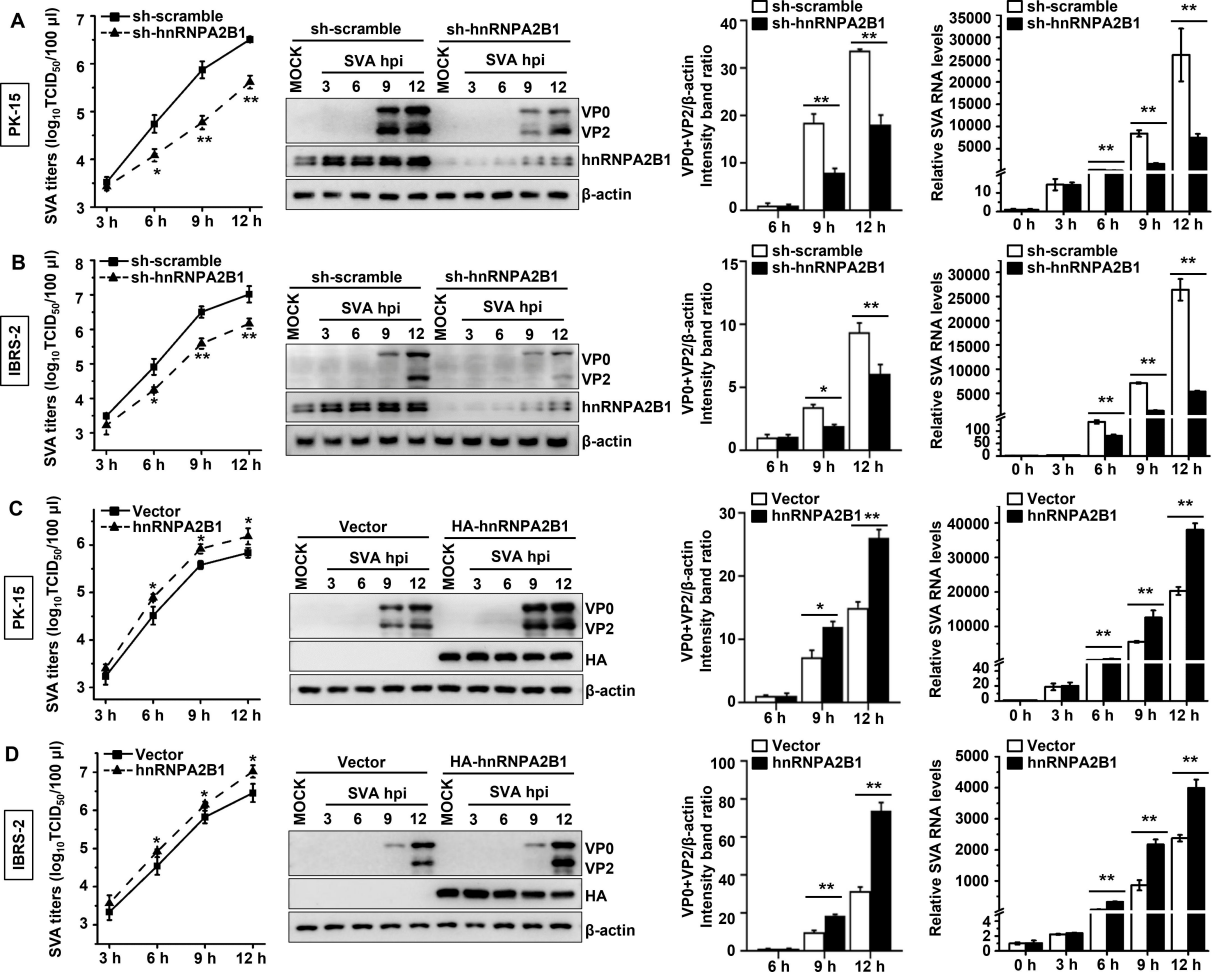
hnRNPA2B1 positively regulates the replication of SVA. (**A and B**) PK-15 or IBRS-2 cells stably expressing control (sh-scramble) or hnRNPA2B1 (sh-hnRNPA2B1) targeting shRNAs were infected with SVA at a multiplicity of infection (MOI) of 1. Cells and supernatant were harvested at specified time points, and viral titers were determined by TCID_50_ assay on IBRS-2 cells. Cell lysates were analyzed for the expression of hnRNPA2B1 and viral proteins (VP0 and VP2) by Western blotting, and the gray intensities of viral proteins were determined. Total RNAs were extracted and subjected to RT-qPCR analysis to determine the levels of viral RNA. (**C and D**) PK-15 or IBRS-2 cells transfected with empty vector or HA-hnRNPA2B1 were challenged with SVA (MOI of 1), and cell lysates and supernatants were harvested at the indicated times. Viral titers were determined by TCID_50_ assay, and the expression levels of viral proteins and RNA were determined by Western blotting and RT-qPCR, respectively. Data are the means of three independent experiments and error bars indicate standard deviations (SD), *P < 0.05; **P < 0.01.

To determine whether hnRNPA2B1 plays a functional role in the SVA infection cycle, the effect of hnRNPA2B1 overexpression or knockdown on SVA replication in porcine-derived PK-15 and IBRS-2 cells was explored, which are widely used for porcine picornavirus research (37). The ectopic expression or knockdown of hnRNPA2B1 had no significant effect on cell viability, as confirmed by viability assays performed immediately before infection (data not shown). Viral titers were measured to assess the effect of hnRNPA2B1 on SVA replication. Significant reductions in viral titers were observed in hnRNPA2B1 knockdown cells. Viral yields were suppressed by ~4.6-, ~13-, and ~7.8-fold in PK-sh-hnRNPA2B1 cells and by ~4.6-, ~8.2-, and ~7.1-fold in IBRS-sh-hnRNPA2B1 cells at 6, 9, and 12 hpi, respectively. In contrast, viral yields increased in both PK-15 and IBRS-2 cells that ectopically expressed hnRNPA2B1 (Fig. 1, left). Western blot analysis of viral protein production revealed a significant reduction in the expression of VP2 and its precursor, VP0, in hnRNPA2B1 knockdown cells. However, the production of viral proteins displayed a significant increase when hnRNPA2B1 was ectopically expressed (Fig. 1, middle). Finally, the effect of hnRNPA2B1 on viral RNA accumulation was measured using quantitative reverse transcription polymerase chain reaction (RT-qPCR). Consistent with the viral titers and protein yields, a significant reduction in viral RNA synthesis was detected in hnRNPA2B1 knockdown cells, whereas a significant increase was observed after the ectopic expression of hnRNPA2B1 (Fig. 1, right). Collectively, these data demonstrate that hnRNPA2B1 plays a proviral role in SVA replication in both cell types.

### SVA infection stimulates endogenous expression and nucleocytoplasmic translocation of hnRNPA2B1

**Fig 2:**
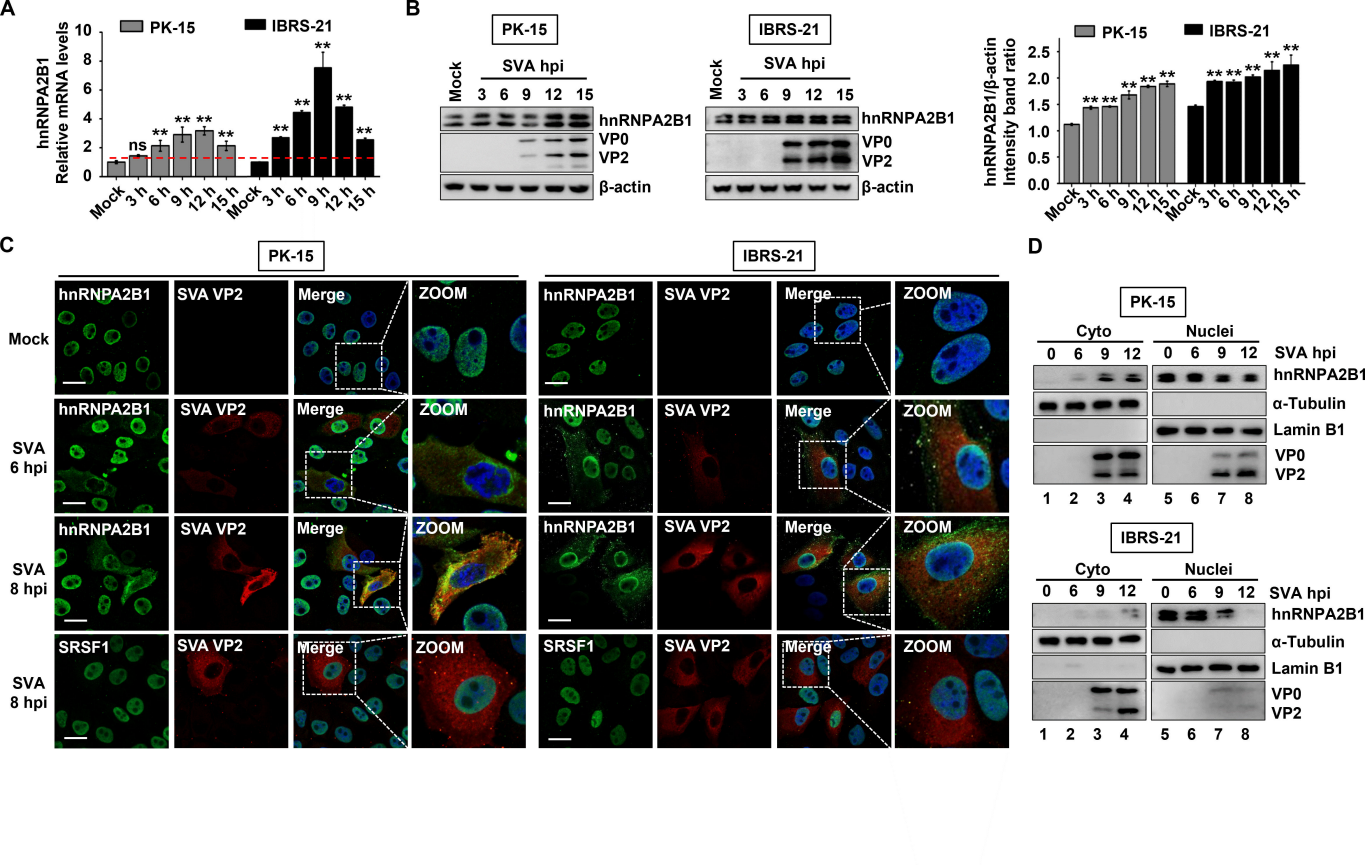
SVA infection upregulates the expression of hnRNPA2B1 and induces translocation of hnRNPA2B1 from nucleus to cytoplasm. (**A and B**) PK-15 or IBRS-2 cells infected with SVA [multiplicity of infection (MOI) of 1] were collected at specified time point. The transcriptional levels of hnRNPA2B1 were determined by RT-qPCR, and the levels of hnRNPA2B1 protein and viral proteins were measured by Western blotting. The relative levels of hnRNPA2B1 protein in each sample after normalizing against β-actin was determined using ImageJ software and plotted in bar graphs. Data are the means of three independent experiments and error bars indicate standard deviations (SD), **P < 0.01. (**C**) PK-15 or IBRS-2 cells were mock- or SVA-infected (MOI of 1) and then fixed at 6 or 8 hpi. The cells were permeabilized and subjected to immunofluorescence assay to analyze SVA VP2 (red) and hnRNPA2B1 (green). Nuclei were counterstained with 4',6-diamidino-2-phenylindole (DAPI) (blue). Cells were imaged using confocal microscopy. Scale bar, 20 µm. (**D**) Nuclear and cytoplasmic fractions from PK-15 or IBRS-2 cells infected with SVA at the indicated times were subjected to Western blotting analysis with the indicated antibodies.

To delineate the dynamic changes in hnRNPA2B1 during SVA infection, PK-15 and IBRS-2 cells were mock-infected or infected with SVA for 3, 6, 9, 12, and 15 h to determine the transcript and protein levels of hnRNPA2B1 at each time point. As shown in Fig. 2A, the mRNA levels of hnRNPA2B1 significantly increased by ~2.1-, ~2.9-, ~3.2-, and ~2.1-fold in PK-15 cells and ~4.4-, ~7.5-, ~4.8-, and ~2.5-fold in IBRS-2 cells at 6, 9, 12, and 15 hpi, respectively. Similarly, the protein levels gradually increased in both PK-15 and IBRS-2 cells following SVA infection (Fig. 2B). These results indicate that SVA infection promotes hnRNPA2B1 expression at both the transcriptional and translational levels as the infection progresses.

hnRNPA2B1 is a shuttling protein, which mainly localizes to the nucleus in uninfected cells, but shuttles to the cytoplasm on picornavirus replication. For example, hnRNPA2B1 has been shown to undergo a dramatic nucleocytoplasmic translocation upon EV71 infection (34). As expected, hnRNPA2B1 was gradually redistributed from the nucleus to the cytoplasm in both PK-15 and IBRS-2 cells after SVA infection, especially at 6 and 8 hpi. However, another nuclear protein, serine/arginine-rich splicing factor 1 (SRSF1), retained its nuclear localization during SVA infection (Fig. 2C). This suggests that the nucleocytoplasmic trafficking of hnRNPA2B1 is a specific and selective requirement for efficient viral replication. The cytoplasmic redistribution of hnRNPA2B1 was also confirmed via nuclear-cytoplasmic fractionation and Western blot analysis. The increased accumulation of hnRNPA2B1 in the cytoplasm of infected cells was observed from approximately 6 hpi and continued to increase as the infection cycle progressed (Fig. 2D). Taken together, these results demonstrated that hnRNPA2B1 migrates from the nucleus to the cytoplasm following SVA infection.

### hnRNPA2B1 is translocated to the cytoplasm in an SVA VP3-dependent manner

**Fig 3:**
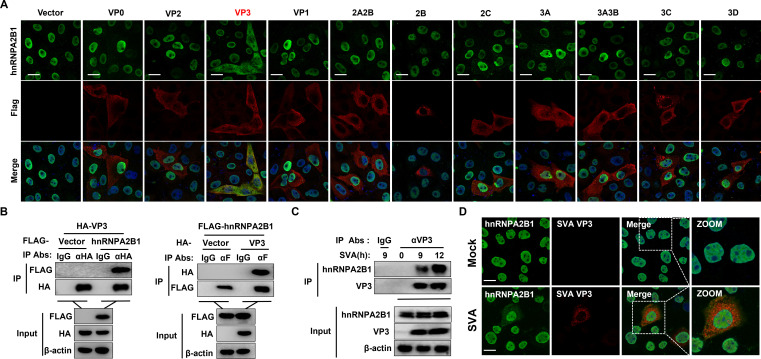
hnRNPA2B1 is translocated to the cytoplasm in an SVA VP3-dependent manner. (**A**) PK-15 cells were transfected with various plasmids expressing SVA structural and non-structural proteins. At 24 h posttransfection, the cells were fixed and analyzed for FLAG-tagged viral proteins (red) and hnRNPA2B1 (green) localization by immunofluorescence assay (IFA). Nuclei were counterstained with DAPI (blue). (**B**) HEK293T cells were co-transfected with a hnRNPA2B1-FLAG-expressing vector (4 µg) and a VP3-HA-expressing vector (4 µg). At 24 h posttransfection, the cells were lysed and immunoprecipitated with anti-HA or anti-FLAG antibodies, followed by immunoblotting analysis. (**C**) Endogenous interactions between VP3 and hnRNPA2B1. Lysates from PK-15 cells infected with SVA at the indicated times were immunoprecipitated with rabbit anti-IgG or anti-VP3 antibodies and subjected to Western blotting analysis using the indicated antibodies. (**D**) Colocalization of VP3 with endogenous hnRNPA2B1. PK-15 cells were mock infected or infected with SVA. At 8 h post-infection, cells were fixed and subjected to IFA to detect VP3 (red) and hnRNPA2B1 (green) with rabbit anti-VP3 and mouse anti-hnRNPA2B1 antibodies. Nuclei were counterstained with DAPI (blue). Cells were imaged using confocal microscopy. Scale bar, 20 µm.

To determine whether the nucleocytoplasmic translocation of hnRNPA2B1 is related to the regulation of viral protein expression, the subcellular localization of hnRNPA2B1 in cells ectopically expressing individual viral proteins was examined by immunofluorescence. We observed no significant change in the localization of hnRNPA2B1 in cells expressing the SVA structural proteins VP0, VP2, or VP1, or individual non-structural proteins, including 3C protease (3C^pro^). Intriguingly, a profound cytoplasmic redistribution of hnRNPA2B1 in cells expressing the SVA VP3 protein in both PK-15 and IBRS-2 cells was observed (Fig. 3A; Fig. S2). These results suggest that the viral structural protein VP3 is responsible for driving hnRNPA2B1 nucleocytoplasmic translocation.

Next, the molecular mechanism by which SVA VP3 specifically redistributes hnRNPA2B1 to the cytoplasm was investigated. Based on the results above (Fig. 3A), ectopically expressed VP3-FLAG and hnRNPA2B1 were efficiently colocalized in the cytoplasm; therefore, it was investigated whether VP3 interacts with hnRNPA2B1. By performing transient transfection and co-immunoprecipitation (IP) experiments, the VP3-hnRNPA2B1 interaction was confirmed (Fig. 3B). To further determine whether SVA VP3 interacts with cellular hnRNPA2B1 in the context of SVA infection, virus-infected PK-15 lysates were immunoprecipitated with a rabbit anti-VP3 polyclonal antibody and probed for the presence of hnRNPA2B1 with a mouse anti-hnRNPA2B1 monoclonal antibody. hnRNPA2B1 was readily detected in SVA-infected cells (Fig. 3C), indicating that endogenous VP3 could interact with hnRNPA2B1 in SVA-infected cells. To confirm that the colocalization was due to the interaction between endogenous VP3 and hnRNPA2B1, PK-15 cells were infected with SVA for 8 h and examined by confocal microscopy. Confocal images of cells immunostained with anti-VP3 and anti-hnRNPA2B1 antibodies confirmed the colocalization of SVA VP3 with hnRNPA2B1 (Fig. 3D). Together, these results demonstrate that SVA VP3 interacts with hnRNPA2B1 and drives its nucleocytoplasmic translocation during SVA infection.

### The RGG box within the C-terminus of hnRNPA2B1 is required for its interaction with SVA VP3 and nucleocytoplasmic translocation

**Fig 4:**
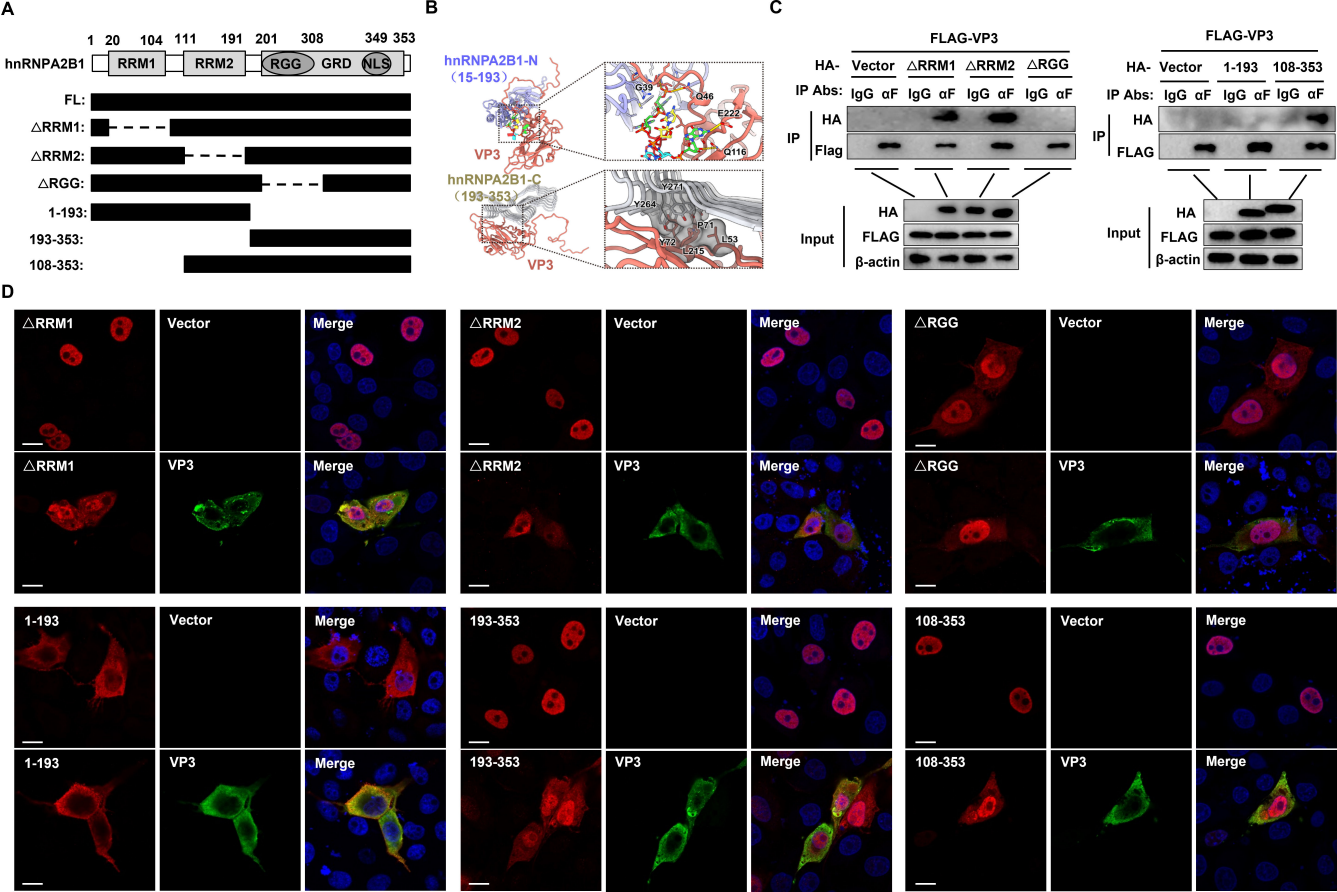
SVA VP3 interacts with the RGG domain at the C-terminus of hnRNPA2B1 and drives its nucleocytoplasmic translocation. (**A**) Schematic diagrams showing the deletion or truncated mutants of hnRNPA2B1 used in Fig. 4B through D. (**B**) Docking results for hnRNPA2B1-N (aa15-193) (blue) or hnRNPA2B1-C (aa193-353) (gray) with VP3 (orange) using ZDOCK. (**C**) Western blotting analysis of immunoprecipitations of cells co-transfected with an expression plasmid encoding each of the indicated mutants of HA-hnRNPA2B1 (4 µg) and a VP3-FLAG-expressing vector (4 µg). (**D**) PK-15 cells were transfected with an empty FLAG vector or VP3-FLAG-expressing vector (1 µg) together with each of the indicated mutants of HA-hnRNPA2B1 (1 µg). At 24 h posttransfection, the cells were fixed and analyzed for VP3 (green) and hnRNPA2B1 mutants (red) localization by immunofluorescence assay . Nuclei were counterstained with DAPI (blue). Cells were imaged using confocal microscopy. Scale bar, 20 µm.

The hnRNPA2B1 contains two N-terminal RNA recognition motifs (RRMs) and a Gly-rich C-terminal domain (GRD) which contains an arginine–glycine–glycine (RGG) box, M9 nuclear localization signal, and core prion-like domain (Fig. 4A). To investigate the hnRNPA2B1 domain(s) responsible for its interaction with VP3, docking of the N-terminus (aa15-193; PDB no. 5HO4) or C-terminus (aa193-353; PDB no. 6WQK) of hnRNPA2B1 to VP3 (PDB no. 6ADT) was carried out using the ZDOCK algorithm in Discovery Studio 2018 (Accelrys, CA, USA). The result indicated that viral VP3 may interact with the surrounding residues of the RGG box within the C-terminus of hnRNPA2B1 (Fig. 4B). Next, HA-tagged mutants of hnRNPA2B1 were created with deletions of various domains and expressed along FLAG-tagged VP3 to perform exogenous co-IP experiments. It was determined that the deletion of individual RRM domains (ΔRRM1, ΔRRM2, and aa108-353) of hnRNPA2B1 had no marked effect on its interaction with VP3. However, the deletion of the GRD (aa1-193) or the RGG box (ΔRGG) led to a failed interaction with VP3 (Fig. 4C). These results indicated that the RGG box within the C-terminal GRD domain is necessary for hnRNPA2B1 to interact with SVA VP3.

To investigate the relevance of the interaction between VP3 and hnRNPA2B1 in VP3-mediated cytoplasmic translocation of hnRNPA2B1, subcellular localization of the various hnRNPA2B1 mutants in VP3-transfected PK-15 cells was tested. As shown in Fig. 4D, deletion of an individual RRM domain (ΔRRM1, ΔRRM2, and aa108-353) or two RRM domains (aa193-353) had no apparent effect on the nuclear localization of hnRNPA2B1 in cells co-transfected with a control vector, and partial re-localization of these mutants to the cytoplasm was observed in cells co-transfected with VP3. Intriguingly, the hnRNPA2B1 mutant lacking the RGG box (ΔRGG) was present in both the nucleus and cytoplasm of cells co-transfected with the control vector, and no significant changes were observed in the localization of hnRNPA2B1 ΔRGG in cells expressing VP3. Moreover, hnRNPA2B1 displayed a complete cytoplasmic localization after the deletion of the GRD (aa1-193) in both the control vector- and VP3-transfected cells. Overall, the results indicated that SVA VP3 redistributes hnRNPA2B1 by interacting with the RGG box within the GRD of hnRNPA2B1.

### hnRNPA2B1 positively regulates SVA IRES-driven translation and indirectly affects viral RNA synthesis

**Fig 5:**
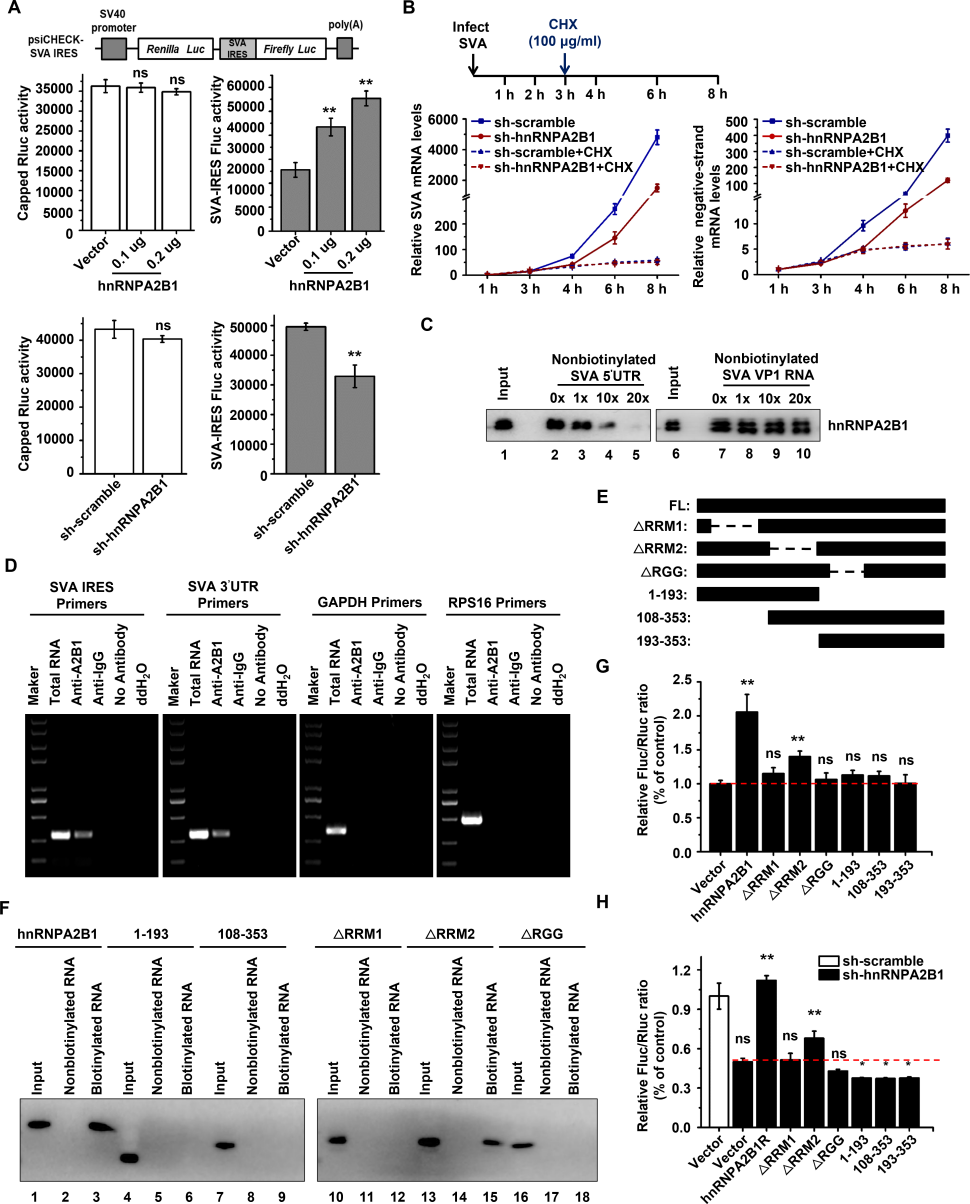
hnRNPA2B1 promotes SVA IRES translation and indirectly affects viral RNA synthesis. (**A**) Schematic illustration of bicistronic SVA IRES construct (psiCHECK-SVA) (top). PK-15 cells treated with the hnRNPA2B1-FLAG-expressing vector with an increasing dose or an empty FLAG vector were transfected with psiCHECK-SVA (200 ng). sh-scramble or -sh-hnRNPA2B1 PK-15 cells were transfected with psiCHECK-SVA (200 ng). At 24 h posttransfection, the RLuc and FLuc activities were determined (bottom). (**B**) sh-scramble or -sh-hnRNPA2B1 PK-15 cells were infected with SVA [multiplicity of infection (MOI) of 1)], and cycloheximide (CHX) (100 µg/mL) was added to the culture medium at 3 h post-infection. RNA was extracted at the indicated times, and the levels of total viral RNA and negative-strand RNA were determined by RT-qPCR. (**C**) Various amounts of unlabeled SVA 5′-UTR (lanes 3–5) and VP1 RNA (lanes 8–10) probes were added to compete with the biotinylated SVA 5′-UTR probe that interacted with hnRNPA2B1. Lanes 1 and 6 contain cell lysate only. (**D**) Cell lysates were generated from PK-15 cells infected with SVA (MOI of 1) at 6 hpi and subjected to an immunoprecipitation assay with an anti-hnRNPA2B1 antibody or isotype anti-IgG antibody. After washing and dissociation, RNA was extracted and subjected to RT-PCR using primers specific to SVA IRES or 3´-UTR, or glyceraldehyde-3-phosphate dehydrogenase (GAPDH), or ribosomal proteins S16 (RPS16). (**E**) Schematic representation of wild-type (WT) hnRNPA2B1 and the indicated mutants used in Fig. 5F through H. (**F**) Cell extracts from PK-15 cells expressing the indicated proteins were collected at 24 h posttransfection and incubated with the non-biotinylated (lanes 2, 5, 8, 11, 14, and 17) or biotinylated SVA 5′-UTR (lanes 3, 6, 9, 12, 15, and 18). The input (lanes 1, 4, 7, 10, 13, and 16) was used to examine protein expression. (**G and H**) PK-15 cells treated with vectors encoding WT hnRNPA2B1 or the indicated mutants were transfected with the bicistronic construct psiCHECK-SVA (**G**). For rescue experiments, sh-hnRNPA2B1 PK-15 cells treated with vectors encoding hnRNPA2B1R (200 ng) or the indicated mutants (200 ng) were transfected with psiCHECK-SVA (**H**). At 24 h posttransfection, the RLuc and FLuc activities were determined. Data are the means ± SD of three independent experiments, *P < 0.05; **P < 0.01.

hnRNPA2B1 must be redistributed from the nucleus to the cytoplasm to facilitate viral RNA synthesis and processing. To determine whether hnRNPA2B1 is involved in SVA IRES-driven translation, the bicistronic luciferase construct psiCHECK-SVA was constructed; it contained cap-dependent *Renilla* luciferase (RLuc) and SVA IRES-dependent firefly luciferase (FLuc) genes (Fig. 5A, top). It was observed that the RLuc activity, which reflects cellular cap-dependent translation, was not significantly affected when hnRNPA2B1 was ectopically expressed or knocked down. However, FLuc activity, which reflects viral IRES-driven translation, was significantly elevated by the ectopic expression of hnRNPA2B1 in a dose-dependent manner and was reduced by the depletion of hnRNPA2B1 (Fig. 5A, bottom). These data demonstrated that hnRNPA2B1 functions as a positive regulator of SVA IRES-driven translation.

During picornavirus infection, viral genomic RNA serves as a template for both translation and RNA synthesis, resulting in the tight coupling of these two processes (38). Hence, it is speculated that the inhibition of translation during infection would lead to reduced accumulation of viral RNA. Consistent with this, a significant reduction in the levels of total and negative-strand viral RNA was detected in hnRNPA2B1 knockdown cells 4–8 h after SVA infection (Fig. 5B). To further dissect whether hnRNPA2B1 has a direct or indirect effect on viral RNA synthesis, the protein synthesis inhibitor cycloheximide (CHX) was used to separate the viral translation and RNA synthesis processes. When there was no measurable difference in the relative amounts of viral replication proteins and progeny RNAs 3 h after SVA infection, blockage of viral translation allowed the measurement of newly synthesized viral RNA only. Notably, no measurable difference in viral RNA levels was observed, regardless of hnRNPA2B1 knockdown (Fig. 5B). These data demonstrate that the effect of hnRNPA2B1 on viral RNA synthesis is indirect and is likely due to the decreased quantity of viral proteins required for genome replication in hnRNPA2B1 knockdown cells.

### RRM1 and RGG domains of hnRNPA2B1 are required for association with the IRES but are not sufficient for IRES-driven translation

Given the functional role of hnRNPA2B1 in viral IRES-driven translation, it was investigated whether hnRNPA2B1 is associated with the SVA IRES. An RNA-protein pull-down assay was carried out with biotinylated RNA containing the SVA 5′-UTR and lysates from SVA-infected cells. Moreover, increasing amounts of non-biotinylated SVA 5′-UTR or VP1 RNA were added to perform a competition assay. As shown in Fig. 5C, the interaction between hnRNPA2B1 and the biotinylated SVA 5′-UTR was out-competed by non-biotinylated SVA 5′-UTR, but not by VP1 RNA, demonstrating their specific association. To further determine whether hnRNPA2B1 associates with the viral genome in infected cells, PK-15 cells challenged with SVA were lysed, and the RNA-protein complexes were immunoprecipitated with an antibody specific to hnRNPA2B1 or isotype anti-IgG. Total RNAs were isolated from these immunoprecipitates and then subjected to RT-PCR using primers specific to either the SVA IRES, 3′-UTR, ribosomal proteins S16 (RPS16), or GAPDH. The results showed that IP with hnRNPA2B1 antibody co-precipitated the SVA IRES and 3′-UTR, but not the control RNAs of RPS16 and GAPDH. No specific RT-PCR band was detected in the immunoprecipitates obtained in the isotype IgG antibody or no-antibody controls (Fig. 5D). Collectively, these results demonstrate that hnRNPA2B1 specifically associates with the SVA genome, both in infected cells and *in vitro*.

To investigate the hnRNPA2B1 domain(s) responsible for its interaction with the SVA 5′-UTR, PK-15 cells were transfected with plasmids expressing various HA-tagged mutants of hnRNPA2B1 (Fig. 5E), and the cell lysates were used for RNA-protein pull-down assays. It was determined that the biotinylated SVA 5′-UTR captured the deletion mutant ΔRRM2, but not the ΔRRM1 or ΔRGG, or the truncated aa1-193 or aa108-353 mutants (Fig. 5F). These results suggest that both the RRM1 domain and RGG box of hnRNPA2B1 are necessary for its interaction with the SVA 5′-UTR.

To investigate the relevance of the interaction between hnRNPA2B1 and the SVA 5′-UTR in driving viral IRES-driven translation, PK-15 cells were transfected with various hnRNPA2B1 mutants and the bicistronic plasmid psiCHECK-SVA underwent subsequent transfection. The results revealed a significant increase in SVA IRES activity stimulated by the ectopic expression of wild-type hnRNPA2B1 and mutant ΔRRM2, but not the other four mutants (Fig. 5G). Furthermore, rescue experiments were performed using a shRNA-resistant mutant, hnRNPA2B1R, and other previously described mutants in sh-hnRNPA2B1 cells. As expected, ectopic expression of hnRNPA2B1R fully reversed the inhibition of viral IRES activity observed upon knockdown, and the ΔRRM2 mutant partially restored IRES activity. However, none of the other mutants were able to restore IRES activity (Fig. 5H). Overall, these findings indicate that the RRM1 and RGG domains of hnRNPA2B1 are essential for its association with SVA IRES but are not sufficient for viral IRES-driven translation, suggesting that both the N- and C-terminal domains are pivotal for hnRNPA2B1-dependent IRES-driven translation.

### Functional interaction of SVA VP3 with hnRNPA2B1 on the 40S ribosome

**Fig 6:**
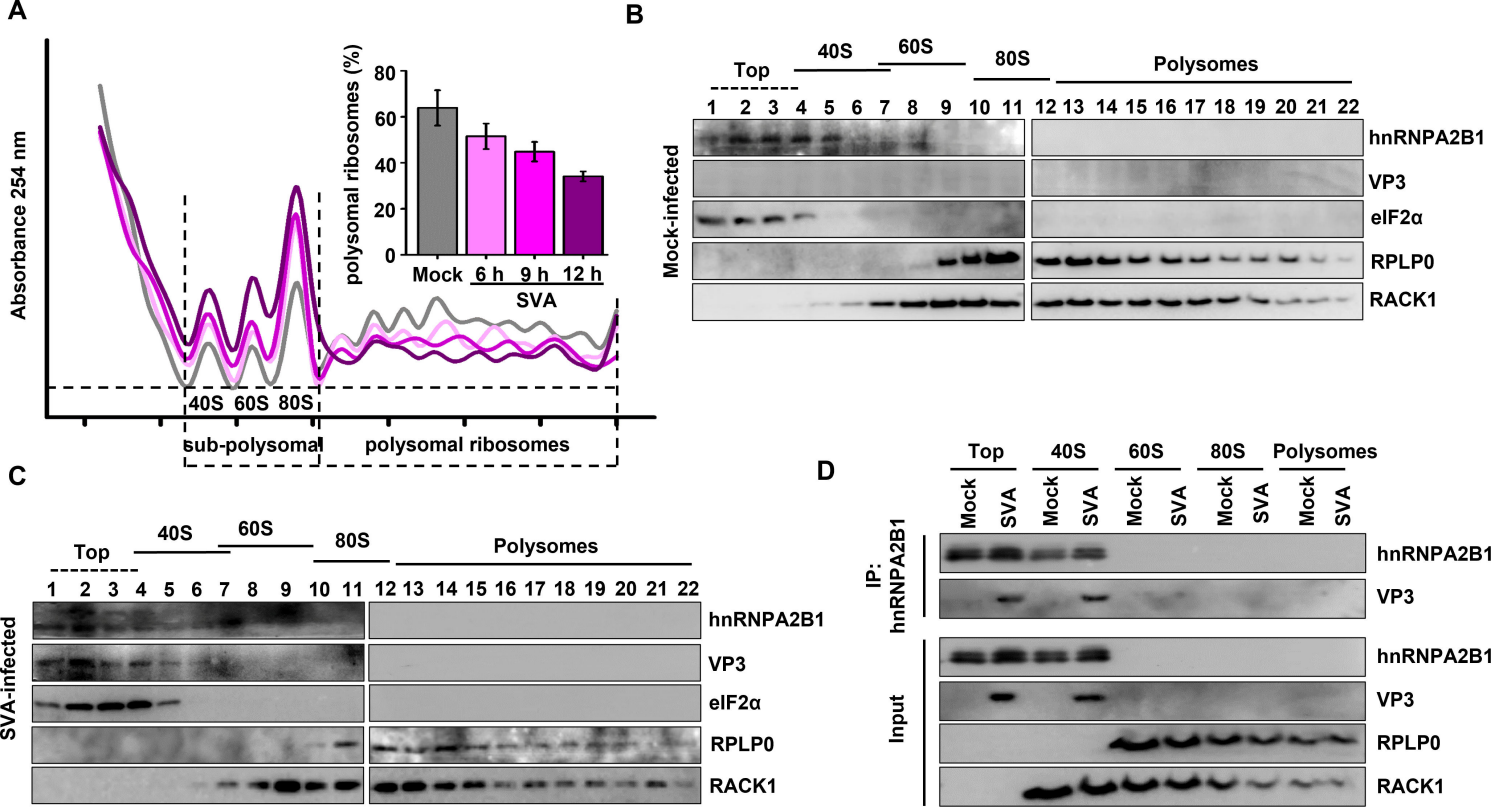
The interaction of SVA VP3 with hnRNPA2B1 takes place on the 40S ribosome. (**A**) Representative polysome profile analysis (lower panel) of mock- and SVA-infected (multiplicity of infection of 1) PK-15 cells at 6, 9, and 12 hpi. The percentage of polysomal ribosomes (actively translating mRNAs) is assessed by measuring the area below the polysomal part of the curve and the area of subpolysomal and polysomal parts of the curve. Histogram bars shown in the upper panel represent the mean percentages of polysomal ribosomes and error bars indicate standard deviations (SD). (**B and C**) Extracts from mock-infected or SVA-infected (9 h post-infection) PK-15 cells were sedimented through 10% to 50% sucrose gradients and fractionated. The collected fractions were then subjected to Western blotting to determine the cosedimentation of VP3 and hnRNPA2B1 with ribosomal subunits (40S and 60S), monosomes (80S), or polysomes. (**D**) Extracts from mock-infected or SVA-infected PK-15 cells were fractionated through sucrose gradients to isolate ribosomal complexes followed by IP with hnRNPA2B1 antibody. The input and the precipitates were analyzed by Western blotting with the indicated antibodies.

To determine if SVA VP3 and hnRNPA2B1 are associated with translation initiation complex-containing fractions and/or actively translating polysomes, a sucrose gradient fractionation of extracts from mock- or SVA-infected PK-15 cells was conducted to separate ribosomal subunits (40S and 60S), monosomes (80S), and progressively larger polysomes. As shown in Fig. 6, the peaks in the polysome profiles were determined by measuring the OD_254_ profiles in the gradient fractions and by probing an immunoblot for RACK1 (40S component) and RPLP0 (60S component). Absorbance monitoring at 254 nm revealed that the proportion of polysomal ribosomes strongly decreased after SVA infection, indicating that cellular bulk translation was compromised by viral infection (Fig. 6A). In extracts from both mock- and SVA-infected cells, hnRNPA2B1 was found primarily at the top of the gradient as well as partially sedimenting in 40S fractions, resembling the sedimentation of eukaryotic initiation factor (eIF)2α, a canonical translation initiation factors (Fig. 6B and C). Moreover, a greater amount of hnRNPA2B1 appeared in the fractions from cytoplasmic extract of SVA-infected cells, and SVA VP3 and hnRNPA2B1 sediment in similar fractions (Fig. 6C). Furthermore, to address whether the functional interaction of VP3 with hnRNPA2B1 is present on the ribosome, various ribosome subunits from lysates of SVA-infected PK-15 cells were isolated, and IP with hnRNPA2B1 antibody was then performed. As shown in Fig. 6D, VP3 co-precipitated with hnRNPA2B1 in the upper and 40S gradient fractions. Taken together, SVA VP3 and hnRNPA2B1 were found, at least in part, in 40S fractions containing viral translation initiation complexes in virus-infected cells, suggesting that there is an interaction between VP3 and hnRNPA2B1 when required for translational control.

### hnRNPA2B1 positively regulates SVA IRES-driven translation initiation

**Fig 7:**
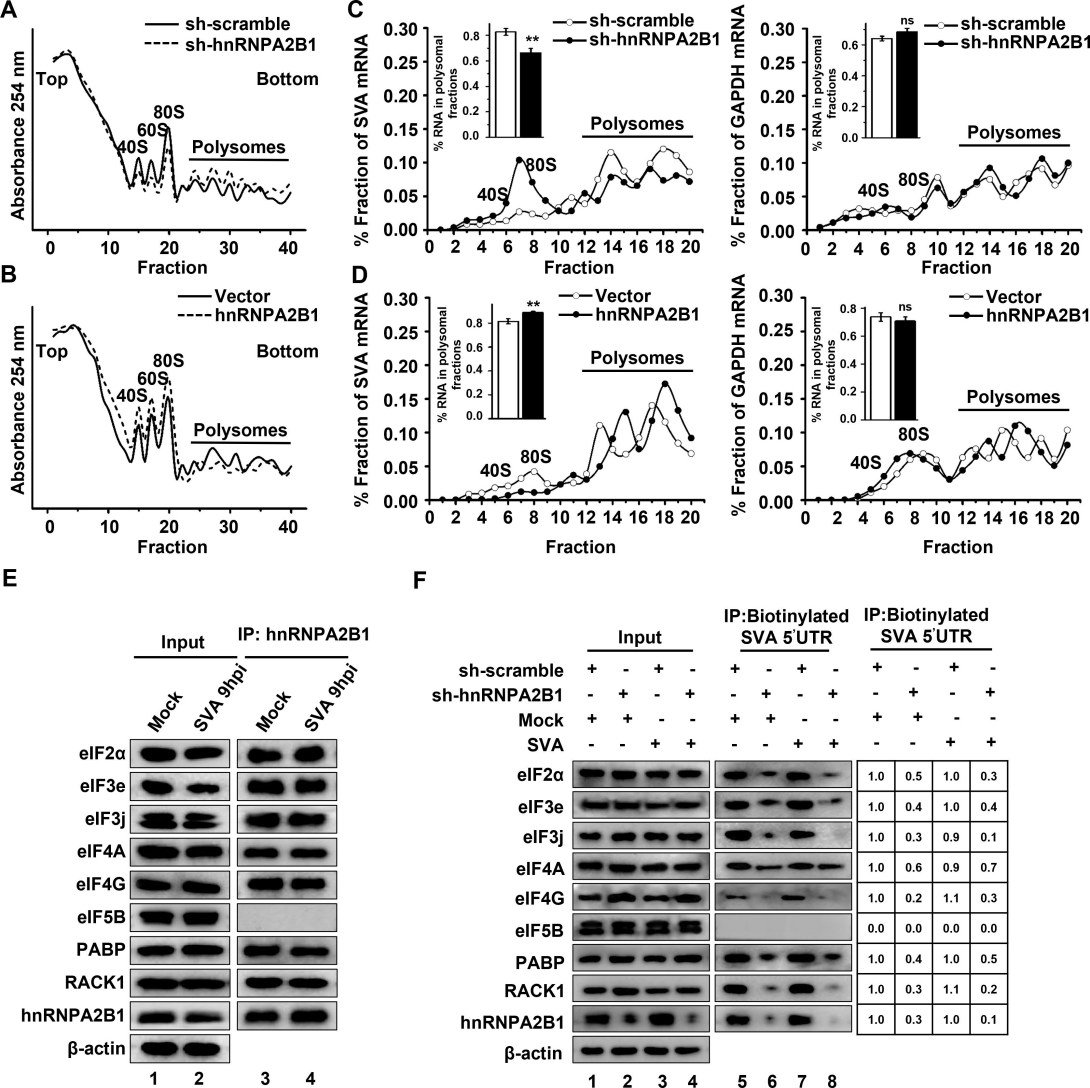
hnRNPA2B1 positively regulates SVA mRNA translation initiation. (A to D) PK-sh-scramble or -sh-hnRNPA2B1 cells were infected with SVA [multiplicity of infection (MOI) of 1]. Alternatively, PK-15 cells were transfected with the FLAG vector or FLAG-hnRNPA2B1 (4 µg) for 24 h and challenged with SVA (MOI of 1). At 9 h post-infection, cell lysates were resolved and fractionated through sucrose gradients. Polysome profiles were obtained by detecting the OD_254_ in the gradient fractions. The fractions were then subjected to RT-qPCR to determine the levels of SVA and GAPDH mRNA. Transcript number is expressed as the percentage of total recovered mRNA transcripts and is plotted against the fraction number. Histogram bars represent the percentages of SVA mRNA and GAPDH mRNA associated with the polysomal fractions. Data are the means ± SD of three independent experiments, **P < 0.01. (**E**) Mock-infected and SVA-infected PK-15 cells were lysed at 9 hpi and then subjected to immunoprecipitation with an anti-hnRNPA2B1 antibody, followed by Western blotting analysis with the indicated antibodies. (**F**) sh-scramble or -sh-hnRNPA2B1 PK-15 cells were infected with SVA (MOI of 1). At 9 h post-infection, cell lysates were collected and subjected to an RNA pull-down assay with the biotinylated SVA 5′-UTR. The input and the precipitates were analyzed by Western blotting using the indicated antibodies, and the gray intensities of these corresponding proteins were also determined.

To gain insights into the mechanism of translational control by hnRNPA2B1, the formation of ribosomal complexes on SVA mRNA was compared to that of the cellular transcript for GAPDH. PK-15 cells that ectopically express or deplete of hnRNPA2B1 were challenged with SVA and the cell extracts were resolved by sucrose density gradient centrifugation (Fig. 7A and B). Following fractionation, the association of ribosomal subunits with specific mRNAs was determined using RT-qPCR. As shown in Fig. 7C and D, the distribution of SVA mRNA shifted to lighter fractions upon hnRNPA2B1 depletion and that the association of SVA mRNA with polysomal ribosomes significantly decreased from 83% to 66%. As expected, the distribution of SVA mRNA shifted to heavier fractions after hnRNPA2B1 overexpression, and the association of SVA mRNA with polysomal ribosomes increased from 81% to 89%. In contrast, there was no obvious effect on the distribution of GAPDH mRNA or its association with actively translating ribosomes when hnRNPA2B1 was knocked down or overexpressed. These experiments establish that hnRNPA2B1 supports the formation of elongation-competent ribosomes on SVA mRNA for effective translation.

The essential function of ITAFs in the IRES-dependent mechanism of translation initiation involves cooperation with initiation factors or the 40S subunit to stabilize the ribosome-IRES association (19). Here, hnRNPA2B1 demonstrated specific coprecipitation with the majority of eIFs, including eIF4G, eIF4A, eIF3e, eIF3j, and eIF2α, but not with eIF5B. hnRNPA2B1 was also associated with polyadenylate-binding protein (PABP) and the 40S component, RACK1 (Fig. 7E). These observations support the hypothesis that hnRNPA2B1 specifically interacts with the translation initiation complexes and promotes the initial step of ribosome binding to the SVA IRES. To further investigate whether hnRNPA2B1 specifically participates in viral IRES-driven translation initiation, a biotin-RNA affinity assay was performed using lysates of mock- or SVA-infected PK-15 cells. It was determined that depletion of hnRNPA2B1 did not affect the expression levels of the indicated eIFs (Fig. 7F, lanes 1–4). Western blot analysis of the pull-down samples demonstrated that eIF4G, eIF4A, eIF3e, eIF3j, eIF2α, PABP, or RACK1, but not eIF5B, could bind to biotinylated SVA 5′-UTR (lanes 5 and 7). Notably, these specific interactions were impaired when hnRNPA2B1 was depleted (lanes 6 and 8). Collectively, these results demonstrate that hnRNPA2B1 supports the binding of translation initiation complexes to the SVA IRES and stimulates viral translation.

### hnRNPA2B1 is involved in the host’s translational shutdown and immune evasion by the SVA VP3 protein

**Fig 8:**
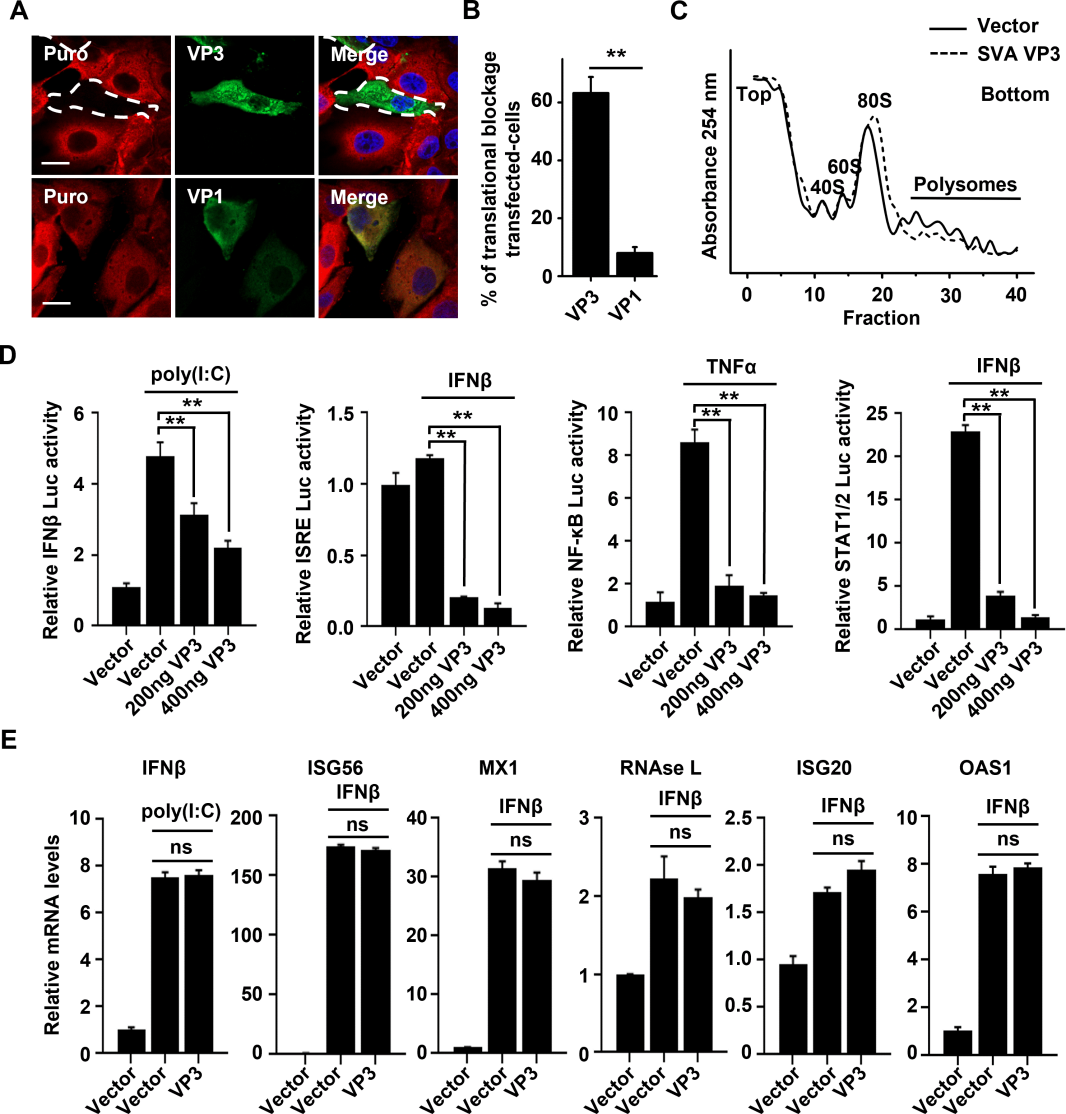
SVA VP3 induces repression of host translation and blockage of host immune response. (**A**) PK-15 cells transfected with the FLAG-VP3 or FLAG-VP1 (1 µg) plasmid for 24 h were incubated with puromycin (5 µg/mL) at 37°C for 30 min. The cells were then fixed, permeabilized, and processed for immunofluorescence assay. A FLAG-specific monoclonal antibody was used to detect SVA VP3 or VP1 (green). Puromycylated chains are visualized using an anti-puromycin antibody (red). Nuclei were stained with DAPI (blue). Scale bar, 20 µm. (**B**) Representative bar plots show the percentage of VP3- or VP1-transfected cells with translational blockage. Total of 100 cells in each group, VP3-positive cells vs VP1-positive cells, were counted in each experiment. (**C**) Polysome profiles of PK-15 cells transfected with an empty FLAG vector or VP3-FLAG-expressing vector (4 µg) were recorded by measuring the OD_254_ of the gradient fractions. (**D**) To assess the effects of SVA VP3 on the induction of firefly luciferase driven by the IFNβ, ISRE, NF-κB, or STAT1/2 promoters, we stimulated HEK293T cells with poly(I:C) (2 µg/mL), IFNβ (500 ng/mL), tumor necrosis factor-alpha (TNFα) (50 ng/mL), or IFNβ (500 ng/mL) as indicated, respectively. Mock treatment was used as a negative control. (**E**) qPCR results for corresponding mRNAs in HEK293T cells transiently transfected with the FLAG vector or FLAG-VP3 and stimulated with poly(I:C) (2 µg/ mL) or IFNβ (500 ng/mL). Data represent the means ± SD of three independent experiments, **P < 0.01.

In addition to favoring viral mRNA translation, viruses might also interfere with the cell’s translation machinery to induce host shutoff, a process that specifically attenuates antiviral responses (9). This study revealed that SVA infection leads to a potent repression of host cell translation (Fig. 6A), and that this process may be dependent on viral-encoded proteins, including VP3 (Fig. 8A; Fig. S3). Intriguingly, a profound absence of puromycin incorporation in cells expressing the SVA VP3 protein was observed, but not in those expressing the VP1 protein (Fig. 8A and B). Furthermore, compared to that of the vector control, the polysome profiles showed a shift from translating polyribosomes to 80S monosomes in the presence of VP3, indicating the global inhibition of translation (Fig. 8C). These results demonstrate that SVA VP3 blocks the bulk translation of host mRNAs that likely encode antiviral factors. Therefore, the effects of SVA VP3 on the interferons (IFN) system were assessed, which is one of the major innate antiviral defense pathways. As expected, the stimulus-triggered expression of firefly luciferase driven by the IFNβ, IFN-stimulated response element (ISRE), nuclear factor-kappaB (NF-κB), or signal transducer and activator of transcription 1/2 (STAT1/2) promoters was effectively reduced by VP3 in a dose-dependent manner (Fig. 8D). However, the expression of VP3 had no significant effect on the mRNA levels of IFNβ, interferon-stimulated gene 56 (ISG56), MX1, RNAse L, ISG20, or OAS1 upon poly(I:C) or IFN-β stimulation (Fig. 8E). This suggests that SVA VP3 acts as a major immune evasion factor through the shutdown of host antiviral gene expression at the translational level, but not at the transcriptional level.

**Fig 9:**
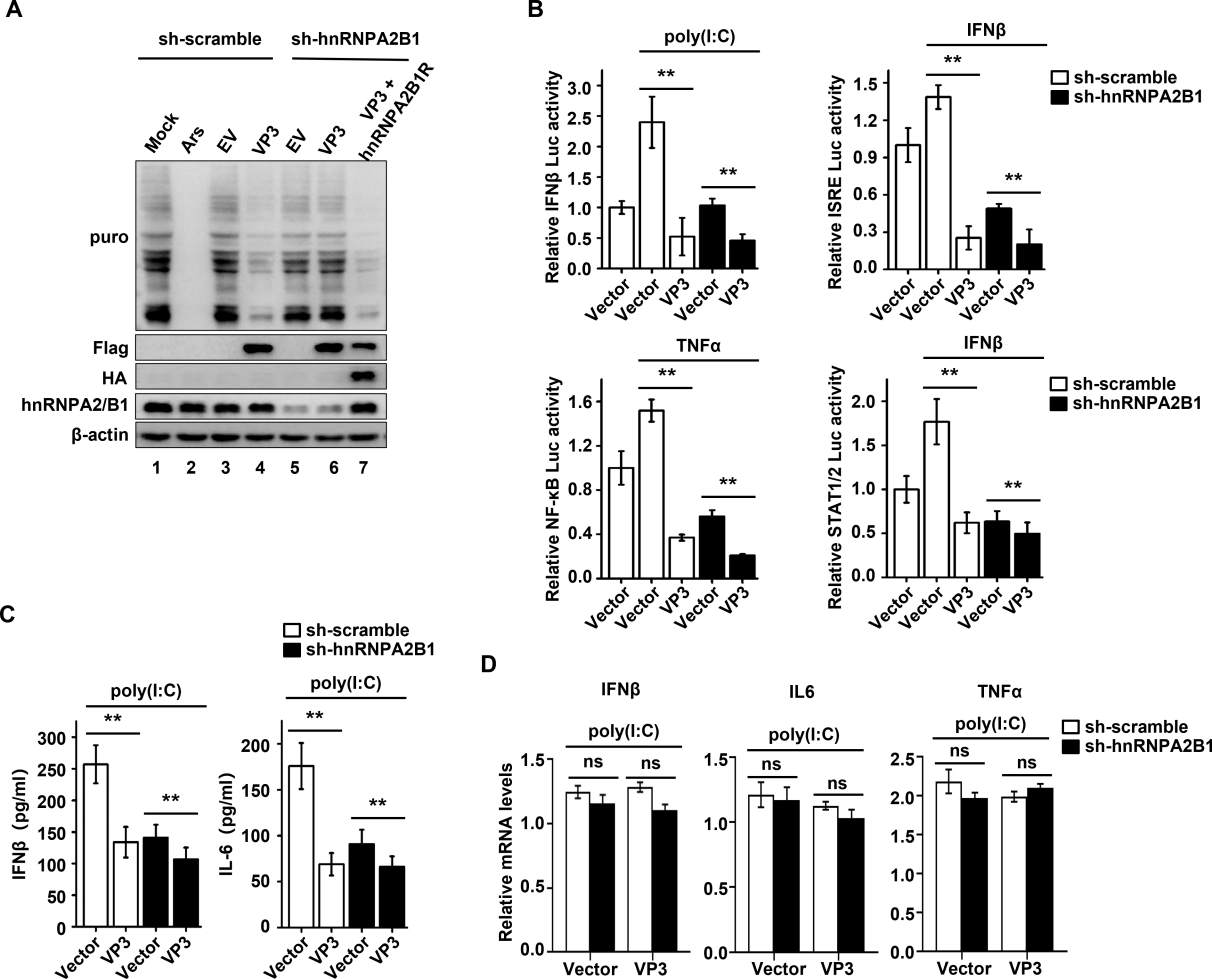
hnRNPA2B1 is specially involved in the effects of SVA VP3 on host translational shutdown and immune evasion. (**A**) sh-scramble and sh-hnRNPA2B1 PK-15 cells were transfected with an empty FLAG vector or a VP3-FLAG-expressing vector (2 µg) together with a vector expressing hnRNPA2B1R (2 µg) in sh-hnRNPA2B1 cells. As a positive control, mock or arsenite (0.5 mM Ars) treatment in sh-scramble PK-15 cells was used for a negative or positive control, respectively. At 24 h posttransfection, cells were incubated with puromycin (5 µg/mL) at 37°C for 30 min, and cell lysates were collected and immunolabeled with the indicated antibodies. (**B**) Relative quantification of IFNβ, ISRE, NF-κB, or STAT1/2 promoter—controlled firefly luciferase activity in sh-scramble and sh-hnRNPA2B1 PK-15 cells transiently transfected with the FLAG vector or FLAG-VP3 and treated with poly(I:C) (2 µg/mL), IFNβ (500 ng/mL), TNFα (50 ng/mL), or IFNβ (500 ng/mL) as indicated, respectively. Mock treatment was used as a negative control. (**C**) Enzyme-linked immunosorbent assay results for IFNβ and IL-6 in the supernatant of sh-scramble and sh-hnRNPA2B1 PK-15 cells transiently transfected with the FLAG vector or FLAG-VP3 and treated with 2 µg/mL poly(I:C) for 9 h. (**D**) qPCR results for the mRNA levels of IFNβ, IL-6, and TNFα in sh-scramble and sh-hnRNPA2B1 PK-15 cells transiently transfected with the FLAG vector or FLAG-VP3 and stimulated with poly(I:C). Data are the means of three independent experiments and error bars indicate standard deviations (SD), **, *P* < 0.01.

Given the importance of the hnRNPA2B1-VP3 interaction for translational control and viral replication, it was investigated whether hnRNPA2B1 specifically participates in the observed effects of SVA VP3 on the host’s translational shutdown and immune evasion. As shown in Fig. 9A, a considerable decrease in global protein synthesis was observed after the expression of VP3 (lanes 1–4), whereas this effect was completely abrogated in the absence of hnRNPA2B1 (lanes 5 and 6). However, expression of the shRNA-resistant mutant hnRNPA2B1R rescued VP3-induced translational repression in sh-hnRNPA2B1 cells (lanes 5–7), implying that this process was hnRNPA2B1 dependent. Next, the effect of hnRNPA2B1 on the host’s innate immune system in response to VP3 was investigated. Notably, compared to that of the control, VP3-induced suppression of luciferase translation driven by the IFNβ, ISRE, NF-κB, or STAT1/2 promoters was much less profound in the absence of hnRNPA2B1 (Fig. 9B). In line with these findings, after stimulation with poly(I:C), the protein levels of endogenous IFNβ and interleukin 6 (IL-6) were drastically reduced in the supernatant of VP3-expressing cells, while this inhibition effect was distinctly impaired when hnRNPA2B1 was knocked down (Fig. 9C). However, the mRNA levels of IFNβ, IL-6, and TNFα induced in VP3-expressing cells was not significantly altered by hnRNPA2B1 depletion (Fig. 9D). Altogether, these results demonstrate that the specific interaction of VP3 with hnRNPA2B1 interferes with the cellular translation machinery, resulting in inhibition of the host’s antiviral immune response.

## DISCUSSION

**Fig 10:**
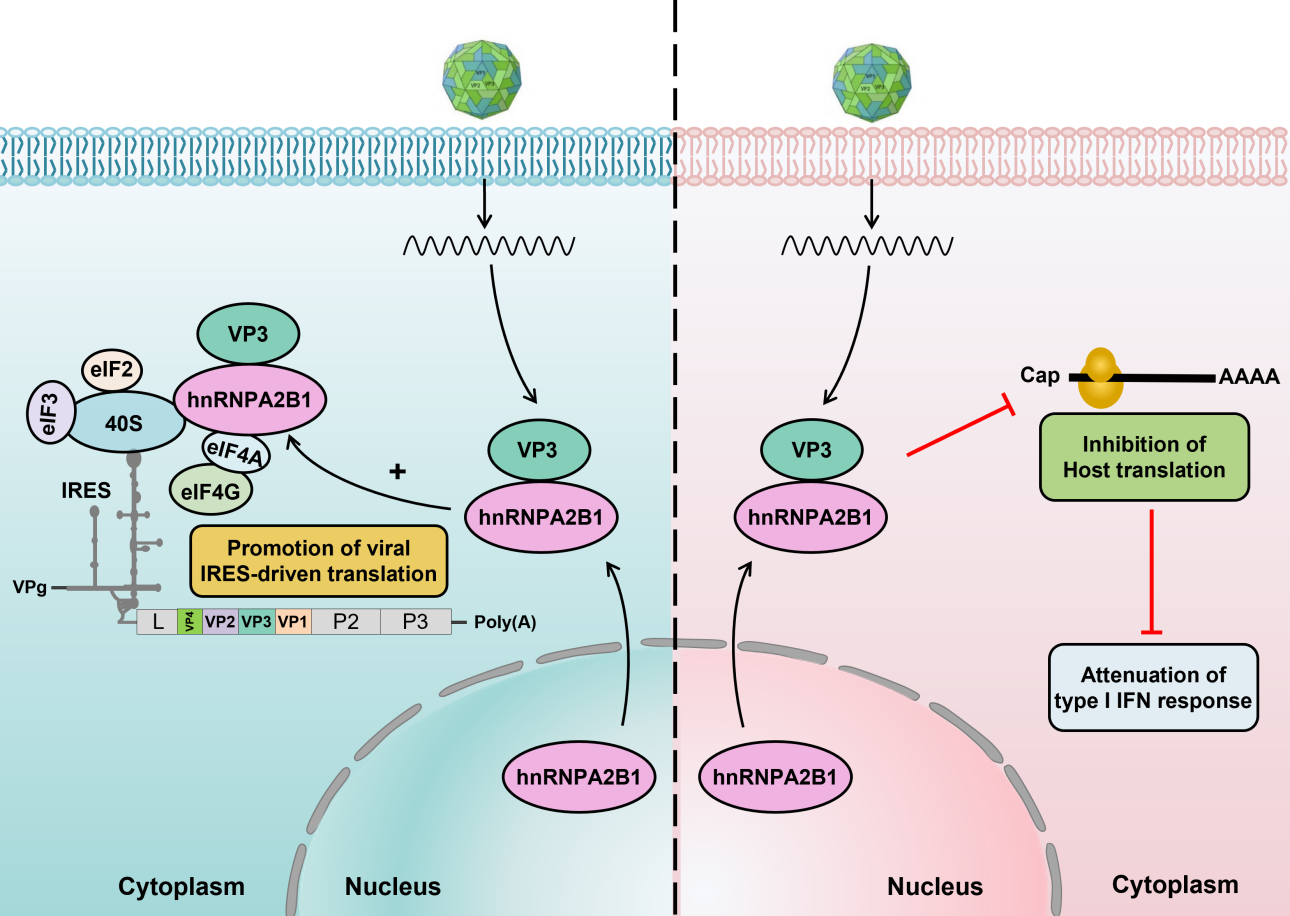
Proposed model of hnRNPA2B1 involved in translation control to facilitate SVA infection. By specific binding to and redistributing hnRNPA2B1 cellular localization, SVA VP3 efficiently subverts host protein synthesis machinery to establish a cellular environment that facilitates viral propagation via a two-pronged strategy: first, hnRNPA2B1, serving as a potent ITAF, is selectively co-opted to promote viral IRES-driven translation by supporting the assembly of translation initiation complexes. Secondly, a global shutoff of host protein synthesis occurs in the context of a VP3-hnRNPA2B1 interaction, resulting in attenuation of cellular innate defenses.

Translational control is crucial during viral-host interactions. Viruses deploy a surprisingly diverse set of strategies to redirect the host’s protein synthesis machinery to repress the host’s mRNA translation while concomitantly allowing the translation of viral mRNAs (9–11). Previous studies have suggested that SVA 3C^pro^ can repress host protein synthesis via cleavage of the translation accessory factor poly(A)-binding protein cytoplasmic 1 (PABPC1) and therefore promote viral replication (39). However, the interwoven interactions between the translation machinery, SVA, and innate immunity are still not fully understood. Here, hnRNPA2B1 was identified and validated as a critical target for manipulating the translational landscape of SVA-infected cells, thereby contributing to the selective prioritization of viral mRNA translation. By binding to and redistributing the cellular localization of hnRNPA2B1, SVA VP3 induces translational reprogramming using a two-pronged strategy: first, hnRNPA2B1, which serves as a potent ITAF, is co-opted to promote viral IRES-driven translation by supporting the binding of translation initiation complexes to the SVA 5′-UTR. Secondly, the interaction of VP3 with hnRNPA2B1 effectively inhibits host protein production, leading to reduced expression of host antiviral response genes and the establishment of a cellular environment that favors viral translation and replication (Fig. 10). The present study defines a previously unobserved role for hnRNPA2B1 in the translational regulation and immune evasion of virus-infected cells.

Accumulating evidence suggests that picornaviruses can subvert the cellular translation machinery and shut down host protein synthesis in infected cells, while promoting viral protein translation in an IRES-driven manner (40–44). Picornaviruses utilize several fundamental strategies to manipulate ITAFs into efficiently driving IRES-driven translation, including proteolytic cleavage, post-translational modification, and disruption of the nucleocytoplasmic trafficking of ITAFs (19, 23, 26, 45, 46). In the present study, it was determined that hnRNPA2B1 undergoes a profound nucleocytoplasmic translocation upon SVA infection, which is in concordance with previous observations following EV71 infection (34). Unexpectedly, the redistribution of hnRNPA2B1 was not dependent on the well-known proteinase 3C^pro^, but was exclusively driven by SVA VP3, via its interaction with the RGG box within the GRD of hnRNPA2B1. The RGG motif is characterized by repeated Arg-Gly-Gly intervals interspersed with other aromatic amino acids, allowing it to interact with a broad range of proteins and nucleic acids (47–49). The results of this study suggest that SVA VP3 specifically binds to the RGG box of newly synthesized hnRNPA2B1 in the cytoplasm and blocks its translocation into the nucleus. Conversely, nucleolin, a previously identified ITAF, is redistributed via cleavage by the proteinase SVA 3C^pro^ (23, 50). Taken together, these data suggest some specificity and selectivity in the various mechanisms by which nuclear proteins are redistributed for efficient viral replication.

In general, viral IRES-driven translation is a systematic engineering technique that involves sophisticated regulation and precise interactions among ITAFs, canonical eIFs, ribosomal subunits, and the IRES. During this process, the essential function of ITAFs involves cooperation with initiation factors or the 40S subunit to stabilize the ribosome-IRES association (17, 19, 22). Here, hnRNPA2B1 was identified as an ITAF that positively regulates SVA IRES-driven translation. Furthermore, hnRNPA2B1 appears to be a conserved determinant of the translation and infection of IRES-containing picornaviruses and flaviviruses (Fig. S4). Moreover, several lines of evidence support the proposition that the N-terminal RRMs and RGG box of hnRNPA2B1 may mediate its binding to the SVA IRES, whereas the C-terminal GRD participates in protein-protein interactions, which synergistically induce a proper IRES conformation and support IRES-mediated internal initiation: (i) the RRM1 and RGG domains of hnRNPA2B1 are required for its association with the SVA IRES, but are not sufficient for viral IRES-driven translation (Fig. 5C through H). Consistent with this observation, it is believed that the RGG box mediates RNA-binding activity and plays an important role in binding strength (28, 48, 49). (ii) The functional interaction of SVA VP3 with hnRNPA2B1 partially takes place on the 40S ribosome (Fig. 6). (iii) hnRNPA2B1 co-precipitates with several components (eIF2α, eIF3e, eIF3j, and the 40S subunit) of the 43S pre-initiation complexes (PICs), eIF4G and eIF4A, and these specific interactions are crucial for the recruitment of 43S PICs to the SVA IRES (Fig. 7E and F). And (iv) polysome profile analysis revealed that hnRNPA2B1 is crucial for the formation of elongation-competent ribosomes on SVA mRNAs (Fig. 7A through D). In addition, given its well-known roles in multivalent interactions with RNAs and proteins, hnRNPA2B1 may be recruited to undergo phase separation during the formation of membraneless organelles (51, 52). Thus, whether SVA could take advantage of this to establish a cellular environment that selectively favors viral IRES-driven translation warrants further research.

Host translation shutoff, which is often detected in virus-infected cells, is a common and effective strategy for counteracting host innate immune responses (9, 53). For example, SARS-CoV-2 Nsp1 induces a near-complete shutdown of cellular protein synthesis, leading to profound inhibition of the cell-intrinsic innate immune response to facilitate efficient viral replication (31, 54–56). The NS1 protein, a virulence factor of influenza viruses, suppresses the host’s gene expression by disrupting mRNA nuclear export and subsequent translation, resulting in the downregulation of antiviral responses (57, 58). Here, our work has revealed a previously unobserved role for SVA VP3 in shutting down the translation of IFNs and other proinflammatory cytokines, as well as IFN-stimulated antiviral ISGs, thus attenuating the IFN response. Functional analysis further underscored that hnRNPA2B1 plays an essential role in the translational shutdown and immune evasion by the SVA VP3 protein. These results suggest that antagonizing VP3 function by blocking its ability to bind to hnRNPA2B1 represents a potential means of inhibiting SVA replication, implicating the VP3-hnRNPA2B1 complex as a potential target for the development of modified antiviral or oncolytic reagents.

In conclusion, our study supports a model whereby SVA VP3 efficiently interferes with the cellular translation machinery through its ability to bind to and redistribute hnRNPA2B1, not only promoting viral IRES-driven translation but also resulting in host translation blockage and immune evasion. However, several important questions remain unanswered. For example, it is unknown whether VP3 regulates hnRNPA2B1-mediated phase separation to benefit the localized translation of viral mRNA and, if so, by which molecular mechanisms. In addition, the detailed mechanisms underlying the interaction of VP3 with hnRNPA2B1 in suppressing host protein synthesis require further experimental studies.

## MATERIALS AND METHODS

### Cells and viruses

HEK293T (ATCC#CRL-11268), PK-15 (ATCC#CCL-33), IBRS-2 (ATCC#CRL-1835), and Vero (ATCC#CCL-81) cells were cultured in Dulbecco’s Modified Eagle’s Medium (Solarbio, China) supplemented with 10% fetal bovine serum (Gibco, USA), penicillin (100 U/mL), and streptomycin (100 µg/mL). Cells were maintained at 37°C, 5% CO_2_. SVA strain CH-HuB-2017 (GenBank accession no. MN922286.1) was stored in our laboratory.

### Antibodies and chemicals

Rabbit polyclonal antibodies anti-hnRNPA2B1, anti-HA, anti-lamin B1, anti-α-tubulin, anti-PABP, anti-eIF2α, anti-eIF3e, anti-eIF3j, anti-eIF4A, anti-eIF4G, and anti-eIF5B and mouse monoclonal antibodies anti-hnRNPA2B1, anti-SRSF1, anti-β-actin, anti-FLAG, and anti-RACK1 were purchased from Proteintech (Rosemont, USA). Mouse anti-puromycin monoclonal antibody was purchased from Millipore (MA, USA). Mouse IgG and rabbit IgG were purchased from Beyotime Biotechnology (Shanghai, China). The secondary antibodies, conjugated with horseradish peroxidase, Alexa Fluor 488 (AF488), or AF594, were purchased from Invitrogen (CA, USA). Rabbit anti-SVA VP2 polyclonal antibody was prepared in our laboratory. Rabbit anti-SVA VP3 polyclonal antibody was kindly provided by Dr. Jiangwei Song at Beijing Academy of Agriculture and Forestry Sciences. Puromycin and sodium arsenite were obtained from Sigma-Aldrich (MO, USA).

### Plasmid constructs

To construct the full-length and diverse mutants of hnRNPA2B1, the corresponding cDNAs were amplified from PK-15 cells with conventional RT-PCR. These cDNAs were then constructed into the pCAGGS-N-HA (Addgene, MA, USA) or p3 × FLAG-CMV-14 (Addgene) to generate HA- or FLAG-tagged constructs. Synonymously, mutagenesis of the shRNA sequence of hnRNPA2B1 was performed with QuickMutation Site-Directed Mutagenesis Kit (Beyotime, Shanghai, China) to produce a mutant plasmid of hnRNPA2B1R. The genes of the SVA structural and non-structural proteins were amplified from the cDNA of SVA strain CH-HuB-2017 and subcloned into p3 × FLAG-CMV-14. Bicistronic reporter plasmids were constructed as previously described (59). Briefly, the sequence of SVA IRES (plus 57 bases of ORF) was cloned into the psiCHECK-2 vector (Promega, WI, USA). Reporter plasmids pIFNβ-Luc, pISRE-Luc, pNF-κB-Luc, and pSTAT1/2-Luc were provided by Dr. Hongbing Shu at Wuhan University.

### Generation of hnRNPA2B1 knockdown cells

PK-15 or IBRS-2 cells were transduced with pSUPER-hnRNPA2B1-shRNA or pSUPER-scramble-shRNA viruses for 3 days. Puromycin-resistant cell clones were selected and analyzed by immunoblotting to determine the efficiency of hnRNPA2B1 knockdown. The hnRNPA2B1-shRNA targeting sequence was 5'-GATCCCCGGGCTCATGTAACTGTGAAGATTCAAGAGATCTTCACAGTTACATGAGCCCTTTTTA-3' and 5'-AGCTTAAAAAGGGCTCATGTAACTGTGAAGACTCTTGAATCTTCACAGTTACATGAGCCCGGGG-3'. The scramble-shRNA sequence was 5'-GATCCCCTTCTCCGAACGTGTCACGTTTTTCAAGAGAAAACGTGACACGTTCGGAGAATTTTTA-3' and 5'-AGCTTAAAAATTCTCCGAACGTGTCACGTTTTCTCTTGAAAAACGTGACACGTTCGGAGAAGGG-3'.

### Immunofluorescence assay

PK-15 cells or IBRS-2 cells grown on coverslips were infected with SVA at a multiplicity of infection (MOI) of 1, and at specific times after infection, the cells were fixed with 4% paraformaldehyde. To visualize the subcellular localization of endogenous hnRNPA2B1 and exogenous viral proteins, cells seeded on coverslips were transfected with plasmids expressing FLAG-tagged viral proteins and fixed in 4% paraformaldehyde at 24 h posttransfection. Fixed cells were washed with phosphate-buffered saline (PBS) and permeabilized with 0.1% Triton X-100 for 15 min. Cells were washed three times with PBS and blocked in 1% bovine serum albumin (BSA) for 1 h prior to incubation with primary antibody. Cells were incubated with anti-VP2 (1:300), anti-VP3 (1:300), anti-hnRNPA2B1 (1:300), anti-FLAG (1:300), or anti-puromycin (1:500) diluted in blocking buffer for 1 h followed by three washes with PBS containing 0.1% Tween 20 (PBST). Cells were incubated with the corresponding AF488- or AF594-conjugated secondary antibodies (1:500) for 1 h. Following three washes with PBST, cells were counterstained with 1 µg/mL DAPI (Beyotime) for 10 min, and coverslips were mounted on slides using Antifade Mounting Medium (Beyotime). Cells were imaged using a Zeiss LSM800 laser-scanning microscope (Carl Zeiss, NY, USA).

### Nucleus-cytosol fractionation assay

Nuclear and cytoplasmic fractions extraction of PK-15 or IBRS-2 cells was generated according to the instruction in the Nuclear and Cytoplasmic Protein Extraction Kit (Invent, USA). The cytoplasmic and nuclear fractions were then subjected to immunoblotting with the indicated antibodies. α-Tubulin and lamin B1 were used as the cytoplasmic and nuclear protein markers, respectively.

### Dual-luciferase reporter assay

PK-15 cells in which hnRNPA2B1 was knocked down or overexpressed were transfected with the indicated bicistronic constructs. At 24 h posttransfection, the cell extracts were harvested in a passive buffer and examined for RLuc and FLuc activities in a Lumat LB9507 bioluminometer with the Dual-Luciferase Reporter Assay System (Promega).

HEK293T cells or sh-hnRNPA2B1PK-15 cells were transfected with FLAG vector or FLAG-VP3 in single amounts or increasing amounts, together with pIFNβ-Luc (100 ng), pISRE-Luc (100 ng), pNF-κB-Luc (5 ng), or pSTAT1/2-Luc (100 ng). At 24 h posttransfection, cells were treated with the corresponding stimulus for 9–12 h, and the luciferase activities were then measured with a dual-luciferase reporter assay kit (Promega).

### Co-immunoprecipitation

Cells transfected with plasmids expressing indicated proteins were lysed in M2 lysis buffer [20 mM Tris-HCl (pH 7.5), 0.5% NP-40, 10 mM NaCl, 3 mM EDTA, and 3 mM EGTA] containing protease inhibitors (Beyotime). After sonication for 2 min, the cell lysates were centrifuged at 13,000 × *g* for 10 min at 4°C, and the supernatants were subjected to immunoprecipitation and immunoblotting analysis with the indicated antibodies.

### RNA-binding protein immunoprecipitation and RT-PCR

Lysates from SVA-infected (MOI of 1) PK-15 cells were harvested at 9 hpi and preincubated with protein A-agarose (GE Healthcare, MA, USA) on ice for 1 h. Nonspecific complexes were pelleted by centrifugation at 1,000 × *g* at 4°C for 10 min. An equal quantity of supernatant was mixed with rabbit anti-hnRNPA2B1, control rabbit IgG, or buffer containing no antibody, and incubated on ice for 2 h. The prewashed protein A-agarose was then added to each sample and the samples were incubated on ice for 2 h. The immunoprecipitated RNA-protein complexes were pelleted by centrifugation and washed three times with wash buffer. RNA was then extracted with TRIzol reagent, and RT-PCR analysis was performed with BeyoRTII First Strand cDNA Synthesis Kit (Beyotime) to detect SVA IRES, 3′-UTR, RPS16, and GAPDH gene fragments. The primer sequences are as follows: SVA IRES-F 5′-CTCGACCCTCCTTAG

TAAGGGAA-3′and -R 5′-ATTTGTATGTGCTACCTATAGAAC-3′; SVA 3′-UTR-F 5′- ACAACGACGGCTTATATAAACCAG-3′ and -R 5′-TTCTGTTCCGACTGAGTTCTCCC-3′; GAPDH-F 5′-GTCCATGCCATCACTGCCACCCAG-3′ and -R 5′-GCTGTTGAAGTCACAGGA

CACAAC-3′; RPS16-F 5′-CTGCAGCCATGCCTTCCAAGGGT-3′ and -R 5′-TCATCACGATGG

GCTTATCGGT-3′.

### *In vitro* transcription and biotinylated RNA pull-down assay

SVA 5′-UTR cDNA was amplified from SVA genome and inserted into the pcDNA3.1 vector (Invitrogen) to construct pcDNA3.1-SVA 5′-UTR followed by linearization with BamHI. The transcript of SVA 5′-UTR was synthesized with the RiboMAX Large Scale RNA Production Systems (Promega). SVA 5′-UTR RNA was then conjugated to biotin using Pierce RNA 3' End Desthiobiotinylation Kit (ThermoFisher, CA, USA). Then, the biotinylated RNA pull-down assay was performed with the Pierce Magnetic RNA-Protein Pull-Down Kit (ThermoFisher). Briefly, the biotinylated SVA 5′-UTR (50 pmol) was incubated with streptavidin-conjugated magnetic beads for 30 min at room temperature, and then incubated with cell extract (200 µg) for 60 min at 4°C with agitation. The beads were washed three times with wash buffer and analyzed by immunoblotting with the indicated antibodies.

### Polysome profile analysis

PK-15 cells transfected with indicated FLAG-tagged proteins or sh-hnRNPA2B1 PK-15 cells were infected with SVA (MOI of 1) and incubated with 0.1 mg/mL CHX (Sigma-Aldrich) at 37°C for 5 min to arrest the ribosome at 9 hpi. We then prepared the cell lysates in a buffer containing 20 mM Tris-HCl (pH 7.5), 5 mM MgCl_2_, 100 mM KCl, 1% Triton X-100, 0.1 mg/mL CHX, 50 U/mL RNase inhibitor, and 1 mM phenylmethylsulfonyl fluoride (PMSF), and applied them onto a 10% to 50% sucrose gradient containing the same buffer and centrifuged at 35,000 rpm at 4°C for 3 h in a Beckman SW41 Ti rotor. After fractionating the gradient fractions, we monitored the absorbance from the fractions at 254 nm and extracted the total RNAs for RT-qPCR analysis. Protein in fractions was analyzed by Western blotting. Where indicated, the separated ribosome subunits were desugared and subjected to IP with hnRNPAB1 antibody.

### RT-qPCR

Total RNA was extracted using TRIzol reagent (Invitrogen) and reverse-transcribed into cDNA using the HiScript reverse transcriptase kit (Vazyme, Jiangsu, China). qPCR was performed in triplicate using ChamQ Universal SYBR qPCR Master Mix (Vazyme). The relative abundance of each gene was normalized to GAPDH mRNA levels and determined based on the standard 2^-ΔΔCT^ protocol. The primers used for RT-qPCR are listed in Table 1.

**TABLE 1:** Sequences of primers used for RT-qPCR

Target	Orientation	Sequence
Porcine GAPDH	Forward	5´-ACATGGCCTCCAAGGAGTAAGA-3´
	Reverse	5´-GATCGAGTTGGGGCTGTGACT-3´
Porcine hnRNPA2B1	Forward	5´-GTTGAGCCAAAACGTGCTGTT-3´
	Reverse	5´-TTTCCAGACTGCCTATCAGTA-3´
SVA	Forward	5´-CACCGACAACGCCGAGAC-3´
	Reverse	5´-GAGATCGGTCAAACAGGAATTTGAC-3´
VSV	Forward	5´-AGGGAACTGTGGGATGACTG-3´
	Reverse	5´-GAACACCTGAGCCTTTGAGC-3´
FMDV	Forward	5´-CAAACCTGTGATGGCTTCGA-3´
	Reverse	5´-CCGGTACTCGTCAGGTCCA-3´
CSFV	Forward	5´-AGCCCACCTCGAGATGCTA-3´
	Reverse	5´-CTATCAGGTCGTACTCCCATCAC-3´
Porcine IFNβ	Forward	5´-GTTGCCTGGGACTCCTCAAT-3´
	Reverse	5´-ACGGTTTCATTCCAGCCAGT-3´
Porcine ISG56	Forward	5´-TCCGACACGCAGTCAAGTTT-3´
	Reverse	5´-TGTAGCAAAGCCCTGTCTGG-3´
Porcine OAS1	Forward	5´-CCAACAGGTTCAGACAGCCT-3´
	Reverse	5´-GACGATGTCGATGGCTTCCT-3´
Porcine MX1	Forward	5´-GTCATCGGGGACCAGAGTTC-3´
	Reverse	5´-TCCCGGTAACTGACTTTGCC-3´
Porcine RNAse L	Forward	5´-AGTAAGACGCCCCTGATCCT-3´
	Reverse	5´-TTCAGCAGGAGGGCGAAAAT-3´
Porcine ISG20	Forward	5´-GGTGCTGTGCTGTACGACAA-3´
	Reverse	5´-CGCTGACCCGTGTTCTGTAA-3´
Porcine TNFα	Forward Reverse	5´-TCCACTCGTTCTGTGACTGC-3´ 5´-TCCCAGGTAGATGGGTTCGT-3´
Porcine IL-6	Forward Reverse	5´-AGGGAAATGTCGAGGCTGTG-3´ 5´-TCCACTCGTTCTGTGACTGC-3´

^*a*^ CSFV, classical swine fever virus.

### Enzyme-linked immunosorbent assay (ELISA)

sh-scramble and sh-hnRNPA2B1 PK-15 cells were transfected with an empty FLAG vector or a VP3-FLAG-expressing vector. At 24 h posttransfection, cells were stimulated with 2 µg/mL poly(I:C) for 9 h. Cell culture supernatants were then analyzed for their cytokine concentrations using ELISA kits for the following cytokines: IFNβ (R&D system, CA, USA) and IL-6 (Novatein Biosciences, MA, USA), according to the manufacturer’s instructions.

### Statistical analysis

All data were derived from at least three independent experiments, and are presented as the mean ± SD of the results of three independent biological replicates. Statistical significance was determined using a Student’s *t*-test by SPSS Statistics (IBM Corporation, USA) and assessed based on the *P*-values: *P* < 0.01 (**), *P* < 0.05 (*).

## Data Availability

The authors confirm that the data supporting the findings of this study are available within the article and its supplemental material.

## SUPPLEMENTAL MATERIAL

Supplemental material jvi.01227-24-s0001.pdf Fig. S1 to S4; Table S1.

ASM does not own the copyrights to Supplemental Material that may be linked to, or accessed through, an article. The authors have granted ASM a non-exclusive, world-wide license to publish the Supplemental Material files. Please contact the corresponding author directly for reuse.
