# Supplementary material for: VP3 protein of Senecavirus A promotes viral IRES-driven translation and attenuates innate immunity by specifically relocalizing hnRNPA2B1
Source: J Virol. 2024 Aug 29;98(9):e01227-24. doi: 10.1128/jvi.01227-24 (PMC11406996; doi:10.1128/jvi.01227-24)
Supplement: Supplemental material — Fig. S1 to S4; Table S1. [file jvi.01227-24-s0001.pdf]

Supplementary Materials for

VP3 protein of Senecavirus A promotes viral IRES-driven translation and attenuates innate immunity by specifically relocalizing hnRNPA2B1

Lu Li[1#], Xinwei Li[1#], Han Zhong[1], Mingyang Li, Bo Wan[1,2,3], Wenrui He[1,2,3], Yuhang Zhang[1,2,3], Yongkun Du[1,2,3], Dongjie Chen[4], Wei Zhang[5], Pengchao Ji[1,2,3], Dawei Jiang[1,2,3]*, Shichong Han[1,2,3]*

*Corresponding author

E-mail: hanshichong081@126.com (SCH); jiangdawei1010@126.com (DWJ)

[#]These authors contributed equally to this work.

This PDF file includes Fig. S1, Table S1, Fig. S2, Fig. S3, and Fig. S4 according to the order.

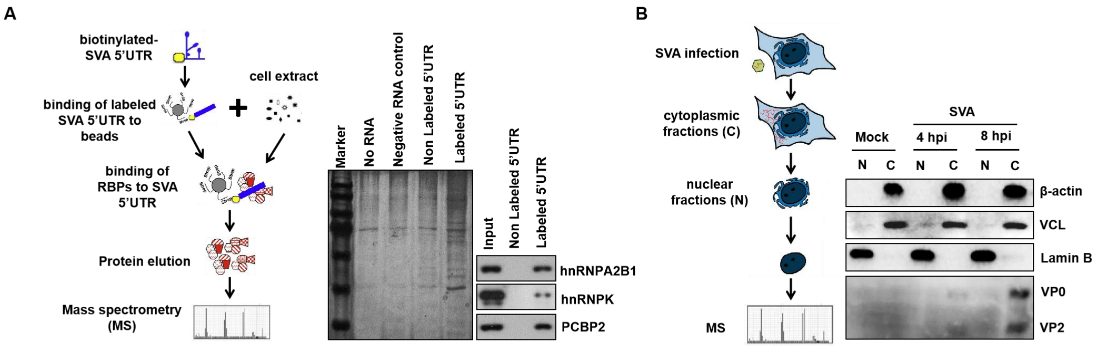

Figure S1. Protocol for screening candidate host RBPs involved in SVA replication.

(A) Procedure summary schematic for identification of cellular proteins associated with SVA 5'-UTR (left). RNA-protein pull-down experiments were performed as described in Materials and methods section. SVA 5'-UTR was labeled with biotin and incubated with streptavidin-conjugated magnetic beads for 30 min at room temperature, and then incubated with PK-15 cell extract for 60 min at 4 °C with agitation. After they had been washed and dissociated from RNA, the eluted proteins were boiled and subjected to Mass spectrometry (MS) or SDS－PAGE (lane 4). Silver staining was applied for visualization. In the negative controls, no RNA was added to the reaction for lane 1; biotin-poly(A)$_{25}$ RNA was added to the reaction for lane 2; non-biotinylated RNA was added to the reaction for lane 3. The associations of specific RBPs with the SVA 5'-UTR (lanes 5 to 7) were then confirmed by Western blotting with the indicated antibodies.

(B) MS analysis of protein distribution. Mock- or SVA-infected PK-15 cells were separated into nuclear and cytoplasmic fractions, digested with trypsin, isotopically labeled, and subjected to LC-MS/MS (left). Fractionation of PK-15 cells following mock, 4, or 8 hours post-SVA infection (hpi) was confirmed by Western blotting analysis (right). Vinculin (VCL) or β-actin was used as a cytoplasmic (C) marker protein and Lamin B as a marker of the nucleus (N). As confirmation of a productive infection, fractions were assayed for the expression of SVA VP2 and its precursor VP0.

Table S1. Identification of several cellular proteins that potentially not only interact with the SVA 5'UTR but also undergo nucleocytoplasmic translocation after SVA infection

Protein name	Protein ID	Mol wt (kDa)	Peptide no.	Sequence coverage (%)	Unused ProtScore	Nuclear abundance ratio of SVA (8 hpi):mock	Cytoplasmic abundance ratio of SVA (8 hpi):mock
**hnRNPA2B1**	A0A4X1TY93	37.429	47	62	238.75	0.82	4.0
**hnRNPK**	A0A8D1XIB0	31.279	19	50.5	311.81	0.84	2.4
**nucleolin**	A0A480LD68	55.849	27	45.8	323.31	0.69	2.5
**hnRNPL**	A0A4X1T159	63.961	13	34.1	168.04	0.72	2.3

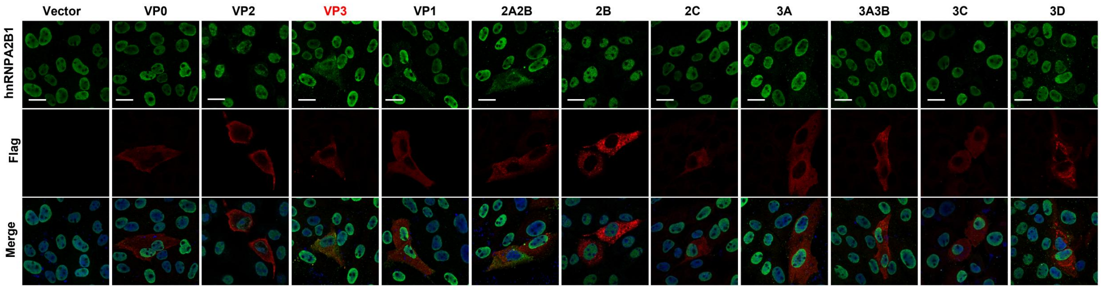

Figure S2. Ectopic expression of SVA VP3 drives nucleocytoplasmic translocation of hnRNPA2B1 in IBRS-2 cells.

IBRS-2 cells were transfected with various plasmids expressing FLAG-tagged viral proteins, VP0, VP2, VP3, VP1, 2A2B, 2B, 2C, 3A, 3A3B, 3C, 3D, or the FLAG vector. At 24 h posttransfection, the cells were fixed and analyzed for FLAG-tagged viral proteins (red) and hnRNPA2B1 (green) localization by IFA. Nuclei were counterstained with DAPI (blue). Cells were imaged using confocal microscopy. Scale bar, 20 μm.

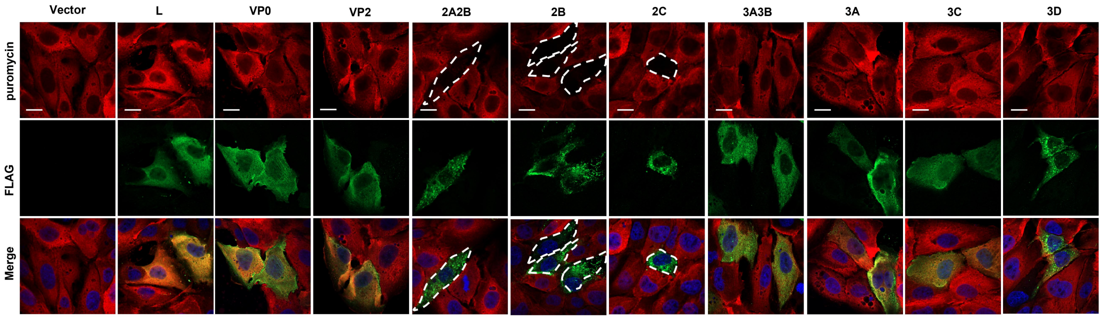

Figure S3. Viral-encoded proteins have a profound impact on the induction of host translation repression by SVA infection.

PK-15 cells were transfected with various plasmids expressing FLAG-tagged viral proteins L, VP0, VP2, VP3, VP1, 2A2B, 2B, 2C, 3A3B, 3A, 3C, 3D, or the FLAG vector. At 24 h posttransfection, the cells were incubated with puromycin (5 μg/ml) at 37°C for 30 min. The cells were then treated and processed for IFA. A FLAG-specific monoclonal antibody was used to detect viral protein (green). Puromycylated chains are visualized using an anti-puromycin antibody (red). Nuclei were counterstained with DAPI (blue). Cells were imaged using confocal microscopy. Scale bar, 20 μm.

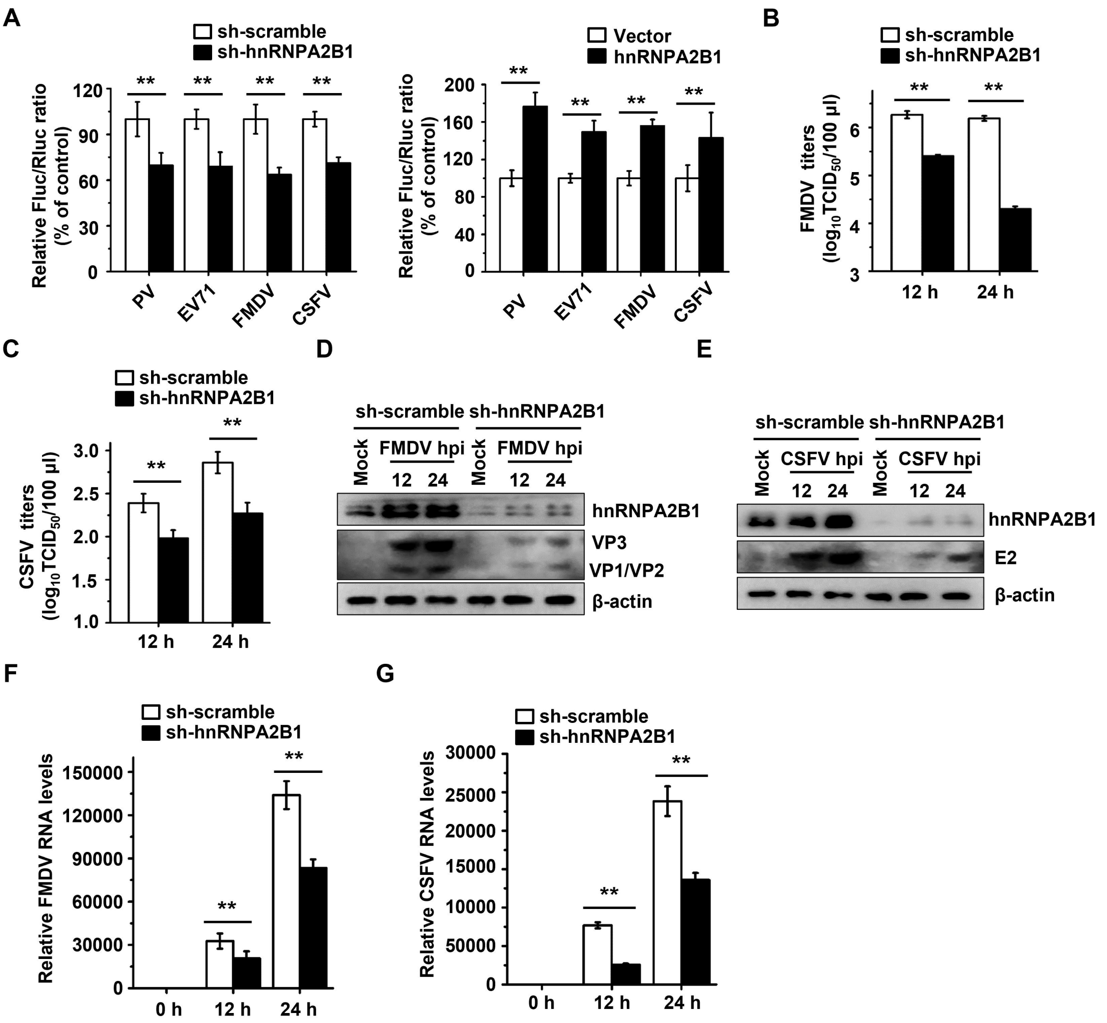

Figure S4. hnRNPA2B1 is a conserved determinant of the translation and infection of IRES-containing picornaviruses and flaviviruses.

(A) sh-scramble or -sh-hnRNPA2B1 PK-15 cells were transfected with the indicated bicistronic plasmid (200 ng) (psiCHECK-PV, -EV71, -FMDV or -CSFV). Alternatively, PK-15 cells treated with the FLAG vector or FLAG-hnRNPA2B1 (200 ng) were transfected with the indicated bicistronic plasmid. At 24 h posttransfection, the RLuc and FLuc activities were determined. Results were normalized to the control (arbitrarily set to 100%) and are presented as means ± SD from three independent experiments.

(B to G) sh-scramble or -sh-hnRNPA2B1 PK-15 cells were infected with FMDV (MOI of 0.1) or CSFV (MOI of 1), respectively. At the indicated times, the supernatants and cell lysates were collected, and virus yields were assessed by $TCID_{50}$ assay (B and C); the expression levels of viral proteins were determined by Western blotting (D and E); total RNAs were extracted and subjected to RT-qPCR (F and G). Data are the means of three independent experiments and error bars indicate standard deviations (SD), ***p* < 0.01.
